# Smart Logistics Model for Supply Chain Management via Brain-Inspired Geometric Deep Networks

**DOI:** 10.3390/biomimetics11060440

**Published:** 2026-06-22

**Authors:** Mehdi Khaleghi, Farshad Pashootanizadeh, Nastaran Khaleghi, Sobhan Sheykhivand, Sebelan Danishvar, VahidReza Ghezavati

**Affiliations:** 1 Department of Industrial Engineering, Islamic Azad University, South Tehran Branch, Tehran 15847-43311, Iran; 2 Faculty of Management, Science and Technology, Amirkabir University of Technology (Tehran Polytechnic), Tehran 15916-34311, Iran; 3 Faculty of Electrical and Computer Engineering, University of Zanjan, Zanjan 45371-38791, Iran; 4 Department of Biomedical Engineering, University of Bonab, Bonab 55517-61167, Iran; 5 College of Engineering, Design, and Physical Sciences, Brunel University London, Uxbridge UB8 3PH, UK

**Keywords:** brain-inspired networks, supply chain logistics, healthcare supply chain, hybrid networks, geometric deep learning, smart logistics, supply chain management, particle swarm optimization

## Abstract

Systematic logistics plays a key role in fostering profitable development in supply chains. An intelligent logistics model can help create a more agile, sustainable, and resilient supply chain. In recent years, several brain-inspired deep learning architectures, such as long short-term memory networks, graph neural networks, and convolutional neural networks, have been introduced for intelligent decision-making tasks. From a biomimetic perspective, these models are inspired by biological information-processing mechanisms. Convolutional neural networks reflect hierarchical procedures similar to those in the visual cortex, graph neural networks mimic communication among biological neurons, and LSTM networks are motivated by short-term and long-term memory mechanisms in the brain. Inspired by these biomimetic computational principles, this study proposes a novel hybrid deep learning strategy composed of LSTM, convolutional layers and GraphSAGE geometric layers for smart supply chain logistics management. This strategy enables leveraging information pertaining to LSTM-based long-term dependencies, convolutional local patterns and graph-related hidden connections of the supply chain dataset for intelligent decision-making. The GraphSAGE framework helps with scalable graph learning, which enhances predictive accuracy in the case of unseen data. The optimizer in the proposed methodology performs sequential optimization using the biomimetic particle swarm optimizer and the Adam approach (PSO-Adam), considering the hybrid cost function. The prediction of logistics parameters is investigated using five datasets, including DataCo, Shipping, Smart Logistics, Hospital Supply Chain, and Pharmaceutical Supply Chain. The average accuracies of 97.8%, 100%, 96.6%, 98.7% and 99.4% are obtained for practical multi-category logistics parameter forecasts. The evaluation metrics for ten logistics predictions confirm the effectiveness of the proposed intelligent logistics model and highlight the potential of biomimetic geometric networks for complex supply chain decision-making. The model is a cost-efficient approach with consideration of the prediction capabilities, helping to reduce the occurrence of logistics risks, increase the productivity of the supply chain and affect the supply chain visibility, customer satisfaction, and industry reputation.

## 1. Introduction

The management processes related to transportation amenities construct logistics connections. Logistics plays a key role in providing sustainability for the supply network. An effective logistics network helps to reduce operational costs and brings competitive advantages to companies. The efficiency of this network and the quality of transportation infrastructure are affected by some factors, including the planning of shipment patterns, the allocation of resources, the agents, the outcomes and the availability of throughput. The demand fluctuations and lack of liability to the external environmental factors lead to a variation in the pattern of transportation equipment. The predictions considering these fluctuations would not correspond to the actual variables, including the date of scheduled loading, type of products, number of loading trains and loading zones. The optimization process is a compulsory element of administering the quality of logistics and management of fluctuations. Applying the optimization approach requires determining the goals of the logistics and the invested timelines. The logistics process considers the interconnection between the key elements of the administration systems of transport services and their intended activities toward the required services. This approach facilitates the functionality of the supply network to make the interaction of particles more perfect and satisfy the operators [1].

The accurate delivery of materials to the destination and rendering the goods to the customers are the main tasks during logistics management. The main idea of a logistics study is to realize an ideal balance in a supply chain. This is the reason that the planning of optimal routes in a supply chain becomes a necessity in a sustainable supply network. The automatic techniques help to promote the benefits of efficient logistics in a supply network and improve the electronic commercial logistics. It is the fundamental element of making an intelligent transportation system. The automatic approach of logistics administration reduces traffic congestion. In addition, it helps to reduce pollution emissions into the environment [2].

Deep learning is a new emerging approach with widespread usage in different applications of data science, signal, and image processing. These deep neural networks are bio-inspired architectures and are designed based on biological activities of the brain cortex during visual data processing [3,4]. The convolutional neural networks are extracting core concepts from hierarchical signal transmission in the visual cortex and mimic dendritic data computation in this cortex. The artificial intelligence employs these bio-inspired networks to understand complex data [5]. The graph networks have been designed and introduced based on the brain connectivity. The neuronal connections in the brain inspire the creation of these networks. The architecture of long short-term memory (LSTM) networks is based on long-term and short-term memory performance in the human brain. Novel deep learning structures contain more complicated biological principles [6]. This type of learning draws its multilayered architecture from the activity of the human brain. This approach uses connected neuronal layers to process complicated data patterns like the activity of the biological human brain. The learning in these methods is performed by tuning the layer connectivity based on large amounts of data. Like the processing of information in the visual cortex, there are multiple neuronal layers in deep learning structures [5]. These structures enable the model to learn complicated data representations. The foundations of these networks are inspired by biological neurons to learn and extract deep features from data. Artificial intelligence has provided the opportunity to implement smart and bionic supply chain models. A bionic supply chain results in the augmentation of artificial intelligence and the human workforce [7]. The fusion approach of artificial intelligence methods and human decision-making is used in the bionic supply chain and has the potential to expedite the fundamental alterations in business. The artificial intelligence-based supply chain improves the performance of the smart supply chain and creates a resilient and sustainable model [8]. This bionic type of supply chain enables the collaboration between machines and humans to foster a resilient supply chain and optimize supply chain performance [9].

Considering the necessity for creating smart supply chain logistics models and bionic supply chains, an efficient bio-inspired network is improved in this study for automatic logistics management tasks. There is a necessity to provide a multi-task approach to improve logistics sustainability during supply chain management. The AI-based approach for modeling supply chain logistics offers end-to-end surveillance and provides automatic predictions for consumer satisfaction [10]. Regarding the connectivity between product feature vectors, allocated resources and plant locations, it is possible to represent the supply chain as a graph. The graph embedding is a prerequisite stage of graph deep learning. The graph-based deep neural network is influenced by the connectivity between different brain lobes during neuronal activities. It is a brain-inspired approach for creating smart systems with automatic decision-making capability [11]. This approach helps to manage the logistics and related risks. In addition, it will enhance the resiliency of the supply chain logistics network. The graph structure helps to identify the hidden connections in the database for categorization [12,13]. The proposed method employs the graph structure of the data to improve the prediction efficiency. Furthermore, it presents a novel geometric architecture and considers the original data samples for each node of the graph illustration. The features are automatically extracted in each layer of the deep structure. It provides a multi- task approach for automatic management regarding different logistics scenarios in a supply chain. The hybrid graph architecture makes use of the advantages of GraphSAGE to effectively capture local structural information of nodes and its scalability to process large-scale graph data. In addition, it considers the capabilities of the convolutional layer to detect local patterns and LSTM layer to extract non-graph spatial dependencies in data sequences.

The contributions presented in this article can be introduced as follows:

(i) It provides a parallel brain-inspired deep network consisting of convolutional, LSTM and GraphSAGE layers for constructing patterns to highlight differences in categories.
(ii) The suggested brain-inspired approach uses graph embedding of the supply chain database. The hidden connections between feature vectors are used for logistics prediction. These connections are inspired by the functional connectivity between different brain regions. The contribution emphasizes the biomimetic aspect of the proposed methodology.
(iii) The proposed brain-inspired network architecture predicts the logistics delay and provides fundamental assistance for risk management.
(iv) It provides a novel structure for logistics parameter prediction, including logistics shipment, logistics traffic status, logistics shipment status, and logistics delays, according to five benchmark datasets.
(v) It presents an intelligent model for restocking strategy forecasting considering healthcare logistics supply chain datasets.
(vi) It creates a smart multi-task logistics supply chain model with efficient performance in various logistics scenarios regarding five significant supply chain databases.
(vii) The important biomimetic aspect of our approach considers sequential optimization utilizing the particle swarm optimizer and the Adam approach.
(viii) The biomimetic aspects of this study are considering brain-inspired deep networks, brain connectivity-based graph input for the proposed geometric network, training deep learning-based smart networks for supply chain logistics management, and healthcare applications regarding Pharmaceutical Supply Chain and Hospital Supply Chain datasets.

The other sections of this paper are organized as follows. Section 2 explains recent methods of logistics and supply chain management using deep learning. In Section 3, the characteristics of the 5-benchmark logistics supply chain datasets are available in detail. Also, it covers the mathematical basis of brain-inspired graph attention and GraphSAGE to describe their functionality. Section 4 unveils the principles of the proposed hybrid deep learning method according to designated logistics tasks. Section 5 represents and extends the outcomes in terms of different evaluation metrics. The figures, tables and plots provided in this section elaborate on the proficiency of the proposed approach. Section 6 is the final part assigned to conclusions.

## 2. Related Works

The techniques of deep learning have been used in various objectives of supply chain management including the planning stage for prediction of future production demand [14,15,16], the customer order designation in a supply chain [17], supplier selection [18,19], the identification of suppliers [20], the transportation and delivery of the productions to the consumers [1,21,22], the allocation of centers for enterprises to reduce costs with appropriate choice of plant and resource location [23], production management and handling the product returns and refunds from consumers [24]. Some deep learning studies have considered threatening risks along with the supply chain. The recognition of the threatening risks of the supply chain is necessary for supply chain risk management. These risks are associated with various natural disasters, infectious disease pandemic situations, geographical vulnerabilities and financial failures [25].

The attributes of transportation and logistics ensure the proficient movement of products from distributors to the end users. Optimizing the logistics and supply chain management leads to proficiency and satisfactory results [2]. Improving load management, delivery programs and route arrangement are some of the significant processes in improving the supply network internal interactions [14]. The emerging factors of greenhouse gas footprint and fuel consumption emphasize logistics’ importance in a supply chain. The deep learning technologies pave the way for data science researchers to provide some beneficial, cost-efficient programs for optimizing the logistics management in a supply network [26,27,28]. In this section, some deep learning strategies for solving transportation problems and supply chain logistics challenges are reviewed briefly [29,30].

Regarding the war situation and conflicts, the fluctuations and instability of the market are the negative consequences of insufficiencies in logistics policy. It was the reason that Drljavca et al. [21] studied the appearance of illegal commercial logistics and the emergence of crime during the conflicts. The hydrogen supply chain has been studied by Jang et al. [31] in 2024. An algorithm has been proposed by them to model the supply chain, considering the demand fluctuations and the capacity of transportation. The proposed methodology in their article provided an effective resolution for the stochastic programming regarding different logistics scenarios in a supply network.

The low-carbon strategies have been considered in logistics management to reduce the air pollutant emissions in order to achieve a green supply chain. The structural equation modeling was the basis of the algorithm by Fu et al. for green supply chain logistics [22] in 2023. The big data technology has been discussed for decision-making and improving a sustainable supply network by Peng et al. [32] in 2022. The carbon regulatory policies have been considered in the study by Peng et al. for the modeling procedure. The logistics preparation and retailing decisions have been performed through modeling with consideration of the carbon-efficient policies.

The combinations of industrial development operations have been applied to logistics management in the study by Matenga et al. [33] in 2022. These practices by Matenga et al. have been based on blockchain technology for automating the processes between software development and information technology operations. This technology has been a database mechanism that allows transparent information sharing within a business network and stores data in a linked chain of blocks. They contributed to a sustainable digital economy, considering the supply chain for the railcar manufacturer. Their work resulted in blockchain-related cloud manufacturing for producing metal parts for boxed sheets. The real-time analytics of the suggested method in their study showed good performance for quality control, inventory management, and consumer reliability.

Niu et al. explored the location choice issue in logistics, capacity sharing, and collaborative methodology among the producers to overcome the challenges of large demand fluctuations [23]. The assumption in their study was about the shipping of the third-party products to distribution centers, whereas the plant productions could be shipped to customer zones directly or through distribution centers. Sirina et al. [1] in 2021 studied the administration of cargo flows and resource allocation. In their study, the management of freight traffic, cargo flows and the stages of process optimization has been applied to the amenities of transport services in Russian Railways.

The choice of a competent logistics as a decision-making procedure considering multiple standards has been studied by Zulqarnain et al. [34] in 2024. The extended fuzzy sets have been used in their proposed model for the interpretation of the ambiguous and unclear data. The Einstein type of aggregation operator has been considered in their study; however, the accuracy level has not been improved by their approach. The supply chain responsiveness corresponding to the logistics strategy in developing countries has been assessed by Anwer [35] in 2022. The dataset in their study consisted of 212 participants in large manufacturing firms in the Middle East. Their study examined the effects of delivery expeditions on the connection between logistics plans and supply chain efficiency.

The expansion of domestic production has been studied by Chen et al. [36] in 2023. They investigated the impact on the functionality and preservation of a nation’s roadways. The concepts of mathematical modeling considering multi-echelon location issues have been exploited for the U.S. domestic production of N95 filtering face mask respirators. The estimation of domestic manufacturing capability has been acquired in terms of household truckloads and motor vehicle miles during the study. The traffic congestion, preservation costs, fuel consumption, pollutant emissions, and traffic have been recognized as the effects of expanding domestic production.

Sustainable halal logistics and collaborations with halal stakeholders are beneficial for the sustainability of a business. Overseeing the segregation of permissible halal products from non-permissible ones is an important factor to be considered in a sustainable halal supply chain. Non-halal logistic providers should not be utilized for carrying halal materials. The probable contamination should be prevented to achieve sustainable aims [37,38,39]. A criterion has been constructed for the separation of permissible from non-halal products in Malaysia [38,40,41,42].

Fenglin et al. [2] proposed a deep reinforcement learning method for particular cold chain logistics. The global route planning has been performed through the upper layer. The local temperature control regulations have been implemented within the lower part of the network. The suggested method caused an improvement in cargo loss and a reduction in energy consumption. The application of a novel network has been explored by Guo Canbo [2] for urban express delivery. The weather factors and traffic congestion have been incorporated innovatively into the state-space modeling. The model has been applied to a logistics center in Beijing and has improved the distribution efficiency. A rural route optimization approach using Monte Carlo tree search has been proposed by Xin Rongyan et al. [2] based on policy gradient with the aim of finding the shortest distribution path.

AlphaGo’s Monte Carlo tree combined with reinforcement learning has been applied to vehicle planning for delivery scenarios by Zhao Yan [43]. The convolutional layers have been used in the proposed model for the verification of the effectiveness on the Amazon transportation dataset. The urban transportation network proposed by the authors has been applied to New York City’s logistics network.

A meta-heuristic approach has been designed for perishable closed-loop poultry supply chains [44]. The NP-hard specifications of the improved model in this meta-heuristic method have been solved considering genetic algorithms and simulated annealing. This approach by Akbari-Aghghale et al. minimizes the supply chain cost. The verification of their methodology has been performed via twenty-four test problems of varying scales [44].

In this article, we propose a novel brain-inspired hybrid geometric network for logistics supply chain management. In the next section, we explain the database settings and the mathematical background of our proposed method. A summary of the transportation and logistics techniques used in supply chain management is shown in Table 1.

## 3. Materials and Methods

In this section, the details of the 5 databases used in this study are explicated. The DataCo, the Shipping and the Smart Logistics databases are used in this study. Also, 2 healthcare logistics datasets, including the Hospital Supply Chain and the Pharmaceutical datasets, have been analyzed in this article. The mathematical basics of brain-inspired GraphSAGE and graph attention networks will be elucidated to understand how the graph layers work in a graph deep network.

### 3.1. Database Setting

Table 2 illustrates the details of the DataCo dataset [41]. A set of 36,000 transactions of the DataCo global company has been analyzed to cover the 4 types of shipping modes in logistics. The types of transactions, days for shipment (scheduled), days for shipping, benefit per order, sales per customer, latitude, longitude, order item discount rate, order item discount, order item total and order profit per order are the characteristics for each data sample. There are 8 different types of order states, including complete, processing, pending payment, closed, pending, on-hold, suspected fraud, canceled, and payment-review. The target labels are considered the late delivery risk status and shipping mode for the automatic forecast of logistics parameters. The four types of shipping modes, considering standard, first class, second class and same day, would be classified with the four-category classification model. Also, the delivery status would be predicted with the two-category classification model. The target tasks for DataCo are shown in Table 3.

The Shipping database is the second dataset utilized in this research. A set of 5280 logistics transfers in this database has been analyzed in this article. The specifications and targets of this database are available in Table 4 and Table 5, respectively. There are 5 warehouses, 3 different categories for shipment mode and 2 categories for reached time classification.

The third one is the Smart Logistics database. A set of 1000 logistics transfers in this database is investigated in this article. The principles about this dataset are accessible in Table 6 and Table 7, respectively. There are 10 logistics IDs, 3 types of shipment status, 3 types of traffic status and 2 categories of logistics delay for prediction of logistics parameters.

**Figure 1:**
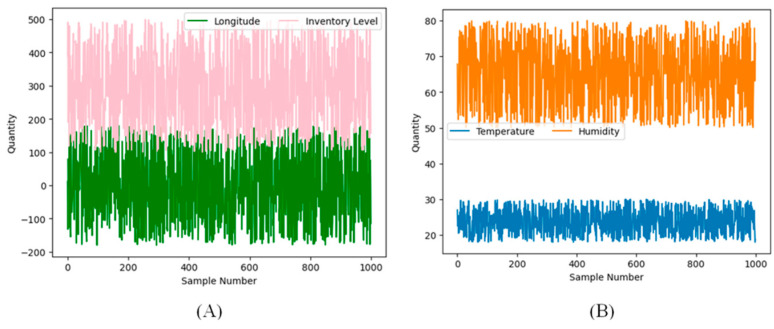
Characteristic plots for the Smart Logistics dataset. (**A**) Longitude, inventory level, (**B**) temperature, humidity.

The graphical illustration of the sample features plays an important role in analyzing the database and providing a model for automatic prediction of the logistics parameters. Figure 1 illustrates the characteristic signals of the Smart Logistics dataset for 1000 samples in this dataset. It shows the fluctuations related to specific characteristics of the Smart Logistics database, including ‘longitude’, ‘inventory level’, ‘humidity’ and ‘temperature’.

**Figure 2:**
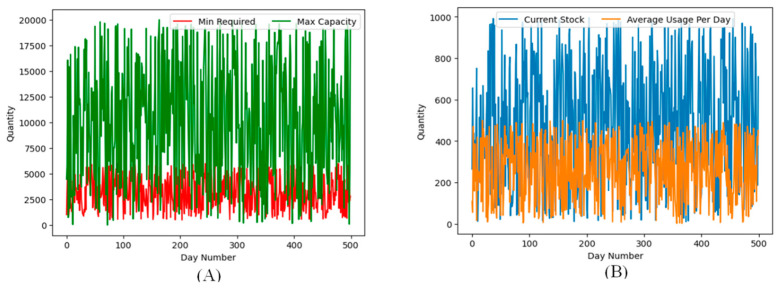
Characteristic plots for the Hospital Supply Chain dataset. (**A**) Min required, max capacity, (**B**) current stock, average usage.

**Figure 3:**
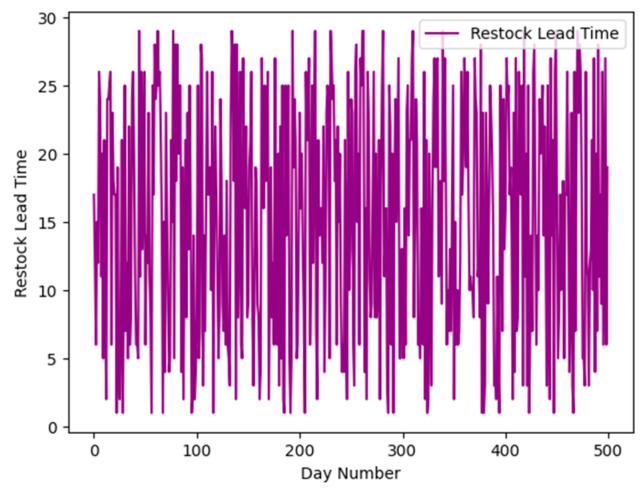
Characteristic plots for the restock lead time parameter of the Hospital Supply Chain dataset.

The features of healthcare logistics datasets are explained in Table 8 and Table 9. The Hospital Supply Chain logistics dataset is analyzed for predicting the restock lead time. Table 8 explains the input and target features of this dataset. The conversion of the text-like features to numerical categories is performed as the pre-processing stage. The restock lead time is the target logistics feature in this supply chain dataset. Figure 2 illustrates four sample features corresponding to the Hospital Supply Chain dataset. Figure 3 demonstrates the target feature fluctuations for logistics prediction regarding this healthcare logistics database.

**Figure 4:**
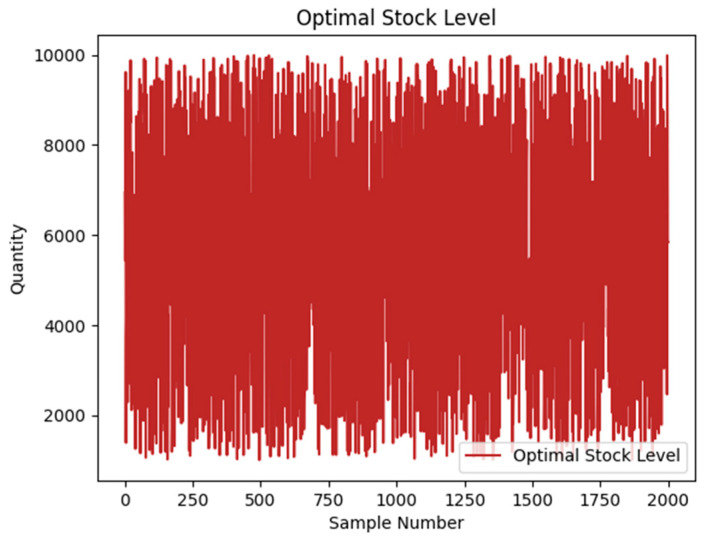
Characteristic plots for the optimal stock level of the Pharmaceutical Supply Chain dataset.

The Pharmaceutical Supply Chain database is the other healthcare logistics dataset in this article. Table 9 explains the input and target features of this dataset. The prediction of the restocking strategy is evaluated with our proposed method. Figure 4 is the graphical illustration of the optimal stock level fluctuations of the Pharmaceutical logistics dataset.

### 3.2. Graph Convolution

The study by Michaël Defferrard et al. [48] was the reason for the improvement of graph signal processing (GSP) applications in different fields of research. The principles of the graph’s particles and the structure of the graph are considered for the mathematical statements in GSP. The GSP employs convolution kernels to enhance the graph domain. The signal processing techniques, like the Fourier transform, are exploited in this area of research and are deployed in graph embedding. Graph spectral filtering is the outcome of using the Fourier transform in GSP, which has been introduced as graph convolution [49]. The brain-inspired graph convolution is introduced by the development of graph convolutional networks regarding the imitation of natural biological interactions and mechanisms to process information. These meta-heuristic strategies are significantly used for parameter tuning of graph convolution networks [48,50].

Considering the graph structure for graph convolution networks, it is compulsory to compute the adjacency matrix. Also, a degree matrix regarding the obtained adjacency matrix is necessary according to the specific graph illustration. The $A\in {ℜ}^{\left(N\times N\right)}$ is the adjacency matrix, and $M\in {ℜ}^{\left(N\times N\right)}$ corresponds to the degree matrix. The calculation of the ***i***-th diagonal component of the degree matrix can be described by (1). The Laplacian matrix of the graph named ***LL*** in the formula is acquired by (2).

(1) $$A_{ii}=\sum_{j}M_{ij}\;$$

(2) $$LL=M-A\in {ℜ}^{\left(N\times N\right)}$$

The fundamental operations in the graph domain are computed in accordance with the eigen vectors of the graph Laplacian matrix denoted by E. These vectors can be obtained via the singular value decomposition (SVD) in (3).

(3) $$LL=E\Lambda E^{T}$$

The columns of $E=[e_{0},\ldots ,e_{N-1}]\in {ℜ}^{\left(N\times N\right)}$ comprise the Fourier basis, and $\Lambda =diag([\lambda_{0},\ldots ,\lambda_{N-1}])$ is a diagonal matrix. Computing the eigenvectors returns the Fourier basis of the graph. For a given signal $X\in {ℜ}^{N}$ designating the accumulated feature vectors on the graph nodes, its graph Fourier transform (GFT) via the output graph basis functions is indicated as (4).

(4) $$\hat{X}=(E^{T})X$$

In Formula (4), $\hat{X}$ denotes the converted signal in the frequency criteria and is the solution correlating with the graph Fourier transform. The above formula describes that the inverse of GFT can be calculated in the form in (5). The filtered form of ***X*** by (***L***L) can be formulated as (6).

(5) $$X=E(E^{T})X=E\hat{X}$$

(6) Y=g(LL)X

Using the following expression in (7), it is clear that the graph convolution of ***X*** with the vector of Eg(***Λ***) is equivalent to the kernel operation of (6). The g(Λ) in phrase (7) is formulated as (8).

(7) $$\begin{array}{l} y=g(LL)x=Eg(\Lambda )E^{T}x=E(g(\Lambda )).(E^{T}x)\\ =E(E^{T}(Eg(\Lambda ))).(E^{T}x)=x{*}_{g}(Eg(\Lambda )) \end{array}$$

(8) $$g(\Lambda )=\left[\begin{array}{ccc} g(\lambda_{0}) & \cdots & 0\\ \vdots & \ddots & \vdots \\ 0 & \cdots & g(\lambda_{N-1}) \end{array}\right]$$

### 3.3. The GraphSAGE Formulation

The embedding part of the GraphSAGE algorithm is described in this section. The denoted variables of K aggregator functions are learned during the training stage. The aggregated information from node neighbors, as well as the set of weight matrices, is utilized to propagate information through the layers of the search depths. Algorithm 1 describes the stages of the GraphSAGE convolutional layer.

According to Algorithm 1, the embedding needs to aggregate information from the representations of the nodes in its neighborhood into a single vector such as $h_{NF(v)}^{k-1}$. The concatenation of the node’s representation with the aggregated vector is performed through the fully connected layer. There is no natural ordering in a node’s neighbors. The aggregator functions in the GraphSAGE algorithm must be able to operate over an unordered collection of vectors. It would be symmetric while still being trainable. The symmetrical property of the aggregator certifies that the model can be employed to randomly order the neighbor feature sets of a node. Three kinds of aggregator functions have been examined, including mean, LSTM and pooling aggregators.

The mean aggregator employs the mean operator. The mean operator is the first candidate aggregator function, and the element-wise mean of the neighboring feature vectors should be considered for the aggregator. This function is similar to the convolutional propagation in the graph convolutional network.

A variant of the graph convolutional network method is explained in Formula (9).

(9) $$\Delta (W.Mean(h_{v}^{k-1}\cup \{h_{j}^{k},\forall j\in NF(v)\})\to h_{v}^{k}$$

The Formula (9) is the customized mean-based aggregator convolutional, and it is a linear estimation of a localized spectral convolution. A more complex function for aggregation in GraphSAGE modeling is the one with an LSTM structure. There is a larger expressive capability for this type of structure. This type of aggregator does not contain a symmetric structure, and it processes the inputs in a sequential mode. The adaptation of LSTMs to operate on an unordered set of neighbors is performed by applying the LSTMs to a random set of neighbors. The pooling method is another type of aggregation as explained in Formula (10). It is symmetric, and it can be trained considering each neighbor’s feature vector. These features are fed using a dense layer, and max-pooling is applied in order to aggregate the information. By employing this operator, various aspects of the neighboring set would be captured. Other symmetric functions, for example, an element-wise mean function, can be used instead of max in Formula (10).

(10) $$Aggregate_{k}^{pool}=\max\left(\{\Delta \left(W_{pool}h_{w_{j}}^{k}+b\right),\forall w_{j}\in NF\left(v\right)\}\right)$$

**Algorithm 1:** The pseudo-code for GraphSAGE.$\begin{array}{l} Input:\\ Graph\;G(\nu (nodes),e(edges));\;input\;feature\;vectors\;\{x_{\nu }\};\;depth\;K;\;weight\;matrices\;W^{k};\\ \Delta nonlinear\;function;\;{Aggregate}_{k},\forall k\in \{1,\ldots ,K\};\;neighboring\;function\;NF:\nu \to 2^{\upsilon }\\ Output:\\ z_{\nu }\;(representation\;vector)\\ x_{\nu }\to h_{\nu }^{0}\\ for\;k=1,\ldots ,K\;do\\ for\;v\in V\;do\\ Aggregate_{k}\;(\{h_{i}^{k-1},\forall i\in NF(v)\})\to h_{NF(V)}^{k};\\ \Delta (W^{k}.Concatenation(h_{v}^{k-1},h_{NF(v)}^{k}))\to h_{v}^{k}\\ end\\ h_{v}^{k}/{\left\|h_{v}^{k}\right\|}_{2}\to h_{i}^{k-1}\\ end\\ h_{v}^{K}\to z_{v} \end{array}$

### 3.4. The Graph Attention

Attention graph networks emphasize the limitations of brain-inspired convolutional graph neural networks by improving self-attention adjustable procedures that specify differing importance to different neighbors [51,52]. These are special technical deep learning structures that accumulate biological attributes such as neuronal interactions into graph-related data processing. These networks use biological information to balance and influence connections between nodes, causing efficient performance for various management tasks.

This section explains and provides formulas for understanding the function of the graph attention layer. A set of features is the input of the graph attention layer as in (11), V and M denote the size of feature vectors and nodes, respectively. A new set of node features would be calculated as the output of the graph attentional layer as in (12).

(11) $$v=\{{\vec{v}}_{1},{\vec{v}}_{2},\ldots ,{\vec{v}}_{M}\},{\vec{v}}_{i}\in R^{V}$$

(12) $$v^{\prime }=\{{{\vec{v}}^{\prime }}_{1},{{\vec{v}}^{\prime }}_{2},\ldots ,{{\vec{v}}^{\prime }}_{M}\},\;{{\vec{v}}^{\prime }}_{i}\in R^{V^{\prime }}$$

The weight matrix $W\in R^{V^{\prime }\times V}$ has been imposed on every single node. The operator of self-attention is utilized to compute the attention coefficients in (13).

(13) $$attention:R^{V^{\prime }}\times R^{V^{\prime }}\to R\;a_{ij}=attention(W{\vec{v}}_{i},W{\vec{v}}_{j})$$

A leaky rectified linear unit can be utilized to compute the normalized output regarding a non-linear activation function as in (14).

(14) aij=exp(Leaky Re LU(w→T[concatenation(Wv→i,Wv→j))∑k∈Niexp(Leaky Re LU(w→T[concatenation(Wv→i,Wv→k))

The normalization procedure employs the first-order neighbor nodes. It is performed across all selections of j, applying the softmax function as delineated in (15).

(15) $${sa}_{ij}=soft\;\max_{j}(a_{ij})=\frac{\exp(a_{ij})}{\sum_{k\in N_{i}}\exp(e_{ik})}$$

The nonlinearity is imposed on the normalized coefficients as in (16). The concatenation of the feature is the next step to create the output as in (17).

(16) $${{\vec{v}}^{\prime }}_{i}=\Delta (\sum_{j\in N_{m}}sa_{ij}W{\vec{f}}_{j})$$

(17) $${{\vec{v}}^{\prime }}_{i}=\overset{K}{\underset{k=1}{Concatenation}}\Delta (\sum_{j\in N_{m}}sa_{ij}^{k}W^{k}{\vec{v}}_{j})$$

For a multi-head attention on the final layer of the network regarding different targets, the averaging function should be considered and the final classification layer should be employed after the averaging stage, as explained in (18).

(18) $${{\vec{v}}^{\prime }}_{i}=\Delta (\sum_{k=1}^{K}\sum_{j\in N_{m}}sa_{ij}^{k}W^{k}{\vec{v}}_{j})$$

This strategy makes the model capable of focusing on important connections to develop the performance of the target prediction by the network. The computational complexity is the negative point of the application of this strategy. These attention graph networks are applicable to abnormality detection in supply chain and logistics management. These networks make the model capable of prioritizing necessary connectivity and achieving accurate investigations.

## 4. Methodology

**Figure 5:**
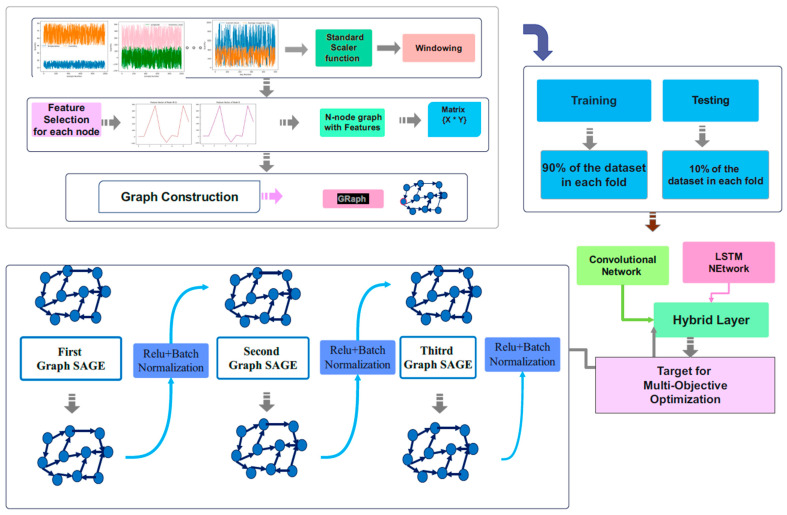
The schematic overview of the proposed H-GSN.

The schematic overview of different stages of the proposed method is provided in Figure 5. The proposed method is employed for logistics forecasts of 5 benchmark datasets, including the Hospital Supply Chain, Pharmaceutical, DataCo, Shipping and Smart Logistics datasets. As can be seen in Figure 5, after the pre-processing and graph embedding stage, the output graph would be applied to adjust the parameters of the proposed hybrid GraphSAGE network (GSN) throughout the training phase. The network consists of three distinct parts of deep networks. The graph-based section includes four sequential layers of the GraphSAGE kernel. The convolutional part includes two sequential non-graph kernel layers, and the LSTM consists of sequential layers. The loss function of the proposed hybrid GraphSAGE network is the weighted summation of the parallel geometric part, the convolutional part of the network and the LSTM section. The training and validation of the H-GSN are performed with K-fold cross-validation.

### 4.1. Pre-Processing Stage

The logistics supply chain datasets in this study are the Hospital Supply Chain, Pharmaceutical Supply Chain, DataCo, Shipping and Smart Logistics datasets. The steps of the pre-processing stage are as follows: zero-one conversion of the binary text-like features, text-to-digit conversion of text features, feature selection, target specification according to different scenarios, cleaning the feature array, balancing the sample numbers regarding each category, standard scaling, and windowing.

1. **Target feature specifications:** We have thoroughly explained the details of this step in the Materials and Methods section (Section 3.1).
2. The text-to-numeric conversions: Converting text-like features in datasets to integers is the initial step of the pre-processing stage. Cleaning the features via the selection of features is another important step. The clean array of features is applied to the graph embedding phase. Setting a balance between sample numbers of different categories during training and classification is another step in the pre-processing stage. The target for training the proposed H-GSN is considered the zero-one conversion of the on-time delivery status and late delivery into zero and one, respectively. Also, the digit conversion of the shipment type has been considered for the DataCo database. For the Shipping database, the target is the logistics shipment modes, the logistics warehouse number, and the binary digit conversion of logistics on-time reaching. The targets regarding the Smart Logistics dataset are logistic IDs, digit conversion of two important logistics parameters, including logistics traffic status and shipment status. The automatic prediction of logistics restocking strategy is the target feature of the two benchmark healthcare datasets.
3. **Sorting and reorganizing the dataset according to the target labels:** This is an important procedure during data preparation for deep learning. The data has been sorted to balance class-specific datasets. For the Mode of Shipment, the dataset has been reorganized and sorted according to the numerical labels. A for loop has been considered in the Python ver 3.13.00 code to reorganize the dataset according to the target label and prevent imbalanced train and test splits regarding each category.
4. **The standard scaling of the feature vectors:** Utilization of min-max scaling and the standard scaling has shown better performance of the prediction strategies considering the standard scaling procedure. It is a necessary part for optimal training of our proposed method. Windowing for constructing a graph of neighboring nodes is another step of the pre-processing stage.
5. The data splitting for cross-validation, train and test splits, has been formed using the scikit-learn package and importing the function of the K-fold strategy.

### 4.2. Graph Construction

After pre-processing, the graph embedding is compulsory to occupy the output graph in the training procedure of the proposed network architecture. The correlation between characteristic features in three databases is essential for graph embedding. A rectified leaky unit is utilized for computing the absolute value of the cross-correlation array.

**Figure 6:**
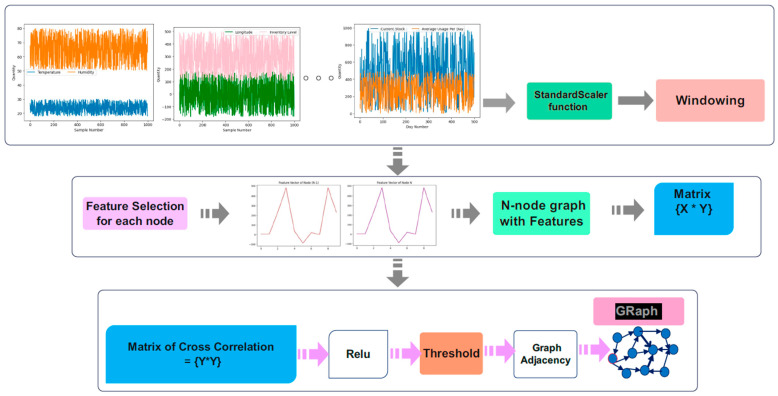
Graph construction stage.

Also, a threshold level is necessary to clean the output array and decrease the computational burden of the algorithm. The adjacency matrix is the result of the leaky rectified linear unit and threshold stage according to the schematic illustration of the graph construction phase in Figure 6.

Deep learning and graph neural networks pave the way to extract some meaningful data from numerical arrays. It is the importance of deep learning to make meaningful feature vectors from the initial data arrays. For example, for data samples of the DataCo dataset, feature correlations capture statistical regularities that are generated by shared latent processes, geographic co-location, correlated types of transactions, correlated types of products, correlated costs of the products, correlated product importance, correlated latitude and longitude of the locations of the suppliers, correlated item types in the healthcare dataset including respiratory equipment, sterilization apparatus, operating room devices or general devices, correlated item types and correlated average usage per day, correlated drug names, and correlated stock levels affected by the usage per day. All of these correlations lead to the graph neural network; all of these correlations have been considered in the graph construction strategy to employ valuable features of graph convolutional layers for the classification of the data samples with highly correlated features. Nodes of the graphs are these data samples, and the node features are the input features that are explained in Table 2, Table 3, Table 4, Table 5, Table 6, Table 7, Table 8 and Table 9. The target features regarding the parameters of supply chain logistics are available in Table 2, Table 3, Table 4, Table 5, Table 6, Table 7, Table 8 and Table 9. There is no target leakage; the target features are just for target variable prediction, and the input features are considered for input graph nodes. This is a graph of data samples with explained node features.

### 4.3. Proposed H-GSN Architecture

**Figure 7:**
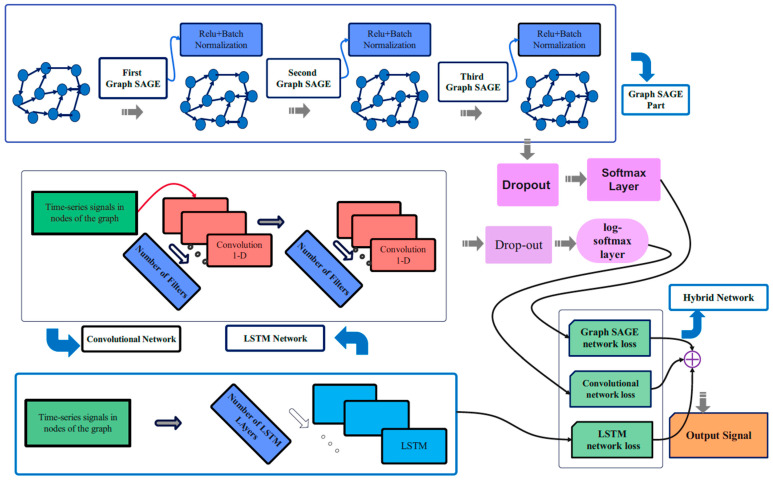
The detailed architecture of the proposed H-GSN.

Figure 7 represents the detailed schematic representation of the proposed network architecture. As this figure shows, our proposed geometric H-GSN contains four layers of GraphSAGE convolution. As designated in this figure, in every GraphSAGE layer, the first step is the estimation of the GraphSAGE of the input graph. The next two important layers are the activation layer and the batch normalization. This kind of normalization makes the network robust and stable during the training procedure, and the speed of the convergence of the network would increase.

The output of the pre-processing stage is imposed on the parallel convolutional part of the hybrid network. The batch normalization is allocated to each GraphSAGE layer. The loss function is the ensemble accumulation of three loss functions of the parallel parts of the H-GSN. After four layers of GraphSAGE and two parallel convolution and LSTM layers, the extracted feature array is acquired with a compatible size regarding the target vector. After log-softmax layers in parallel networks, the obtained signal is classified according to the target vector.

The characteristics of the proposed architecture are explicated with details in Table 10, Table 11 and Table 12. Table 10 explains the details of the GraphSAGE part of the H-GSN regarding two benchmark datasets, including Shipping and Smart Logistics. The details are explained corresponding to two scenarios of logistics shipment mode prediction and logistics ID forecast. Table 11 and Table 12 are the attributes of layers matching the convolutional and LSTM parts of the network according to the above-mentioned scenarios. Also, it shows the kernel size for different layers, the number of kernels, the size of strides and the total number of parameters to be adjusted during the training procedure.

It should be noted that the efficiency of the proposed method is tested within different logistics scenarios. These scenarios are delivery status, warehouse locations, logistics shipping mode, logistics ID and logistics traffic status. The number of categories and the target vectors are different for each scenario. The target vector for delivery status prediction in DataCo is a binary vector. The number of categories regarding the Shipping database is 5 for warehouse locations, 3 for logistics shipping mode and 2 for logistics reaching time. There are 10 logistics IDs, 3 traffic statuses, 3 shipment statuses and 2 logistics delays for logistics prediction scenarios corresponding to the Smart Logistics database. There are three specific categories of logistics restocking strategy regarding two healthcare logistics databases, including the Hospital Supply Chain dataset and the Pharmaceutical Supply Chain dataset. The logistics scenarios regarding the DataCo dataset are 2-dimensional delivery status and 3-dimensional shipment mode prediction.

### 4.4. Training and Evaluation of the Proposed H-GSN

In the training procedure, the input samples along with corresponding targets are utilized to tune the weights and parameters of the proposed H-GSN. A 10-fold cross-validation is performed for training and tuning the variables and parameters of the proposed hybrid GraphSAGE network. The training of the proposed H-GSN is performed according to the detailed parameters regarding network layers in Table 10, Table 11 and Table 12.

**Figure 8:**
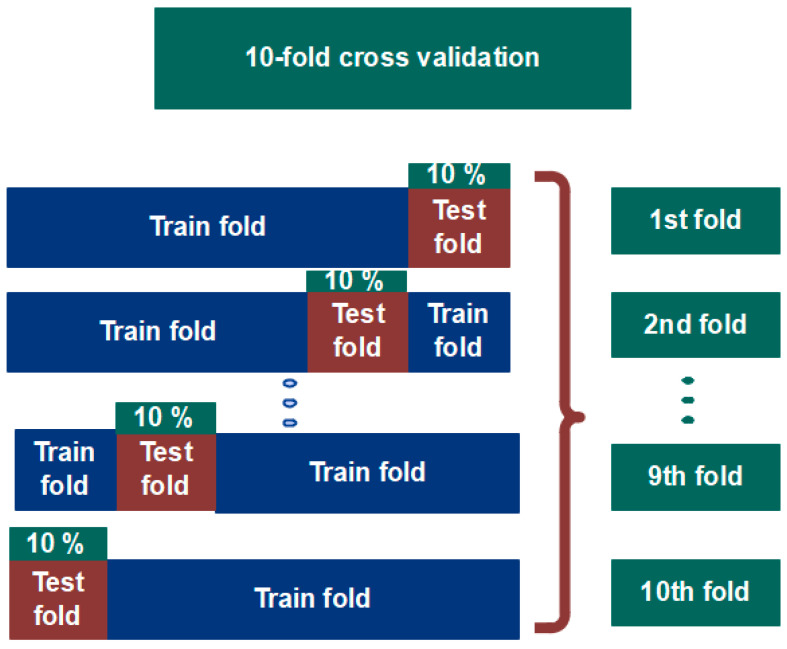
Train and test splits regarding the 10-fold cross-validation stage.

A 10-fold cross-validation is performed using the training samples. The test stage can predict the logistics parameters for 5 benchmark datasets based on the calculated weights of the training stage. The pseudo-code in Algorithm 2 explains the details of the proposed H-GSN. Figure 8 illustrates the training and testing splits in the 10-fold cross-validation stage, which helps catch potential leakage and improves generalization. Also, it helps to achieve temporal separation between training and testing splits. Table 13 describes the parameters of the search space and the optimal value for these parameters during the K-fold cross-validation.
**Algorithm 2:** Pseudo-code for the proposed H-GSN.Proposed Hybrid GraphSAGE Network (H-GSN) **Input:** (1) Data vectors ***X***, (2) A threshold level, (3) Window size for adjacency matrix, (4) Number of layers for parallel parts of the hybrid network, (5) Labeled train and test samples ***Xtrain*** and ***Xtest***, **Output:** Class Labels for X_test_ Initialize the parameters. Training corresponding to the 10-fold cross-validation: 1: Determine the correlation co-efficient of the of ***X*** in ***Xtrain***. 2: Calculate the adjacency matrix ***W*** using the sigmoid function for the result of step 1. 3: Extract the output of the GraphSAGE layers. 6: Calculate the output of the dropout layer. 7: Calculate the output of the parallel convolutional and LSTM layers. 8: Multi-taskoptimization of the weights of the hybrid layers using optimal loss function. 9: Update the weights of the layers regarding the total’s hybrid cost function:
$\begin{array}{l} \left\{\begin{array}{l} Loss_{Cross-Entropy}(t\arg et,output_{1GraphSage})=-\frac{1}{n}\sum_{i=1}^{n}\left(t\arg et_{i}.\log output_{1i}+(output_{1i}-t\arg et_{i}).\log(t\arg et_{i}-real_{i})\right)\\ Loss_{Cross-Entropy}(t\arg et,output_{2Convolution})=-\frac{1}{n}\sum_{i=1}^{n}\left(t\arg et_{i}.\log output_{2i}+(output_{2i}-t\arg et_{i}).\log(t\arg et_{i}-real_{i})\right)\\ Loss_{Cross-Entropy}(t\arg et,output_{3LSTM})=-\frac{1}{n}\sum_{i=1}^{n}\left(t\arg et_{i}.\log output_{3i}+(output_{3i}-t\arg et_{i}).\log(t\arg et_{i}-real_{i})\right) \end{array}\right.\\ Loss_{Total}=Loss_{Cross-Entropy}(t\arg et,output_{1GraphSage})+\beta_{1}Loss_{Cross-Entropy}(t\arg et,output_{2Convolution})+\beta_{1}Loss_{Cross-Entropy}(t\arg et,output_{3LSTM}) \end{array}$ 10: Attain the predictions for the graph illustrations in accordance with ***Xtest*** using the trained H-GSN. Stop specifications: A maximum number of trials or acceptable accuracy.

## 5. Results and Discussion

In this section, the obtained results of verification regarding the proposed H-GSN are presented. Our configuration is executed on a laptop with 16 GB RAM, a GeForce GTX 1050 GPU and a 2.8 GHz Core i7 CPU. The implementation of the proposed network is performed using the Google Colaboratory Pro platform.

**Figure 9:**
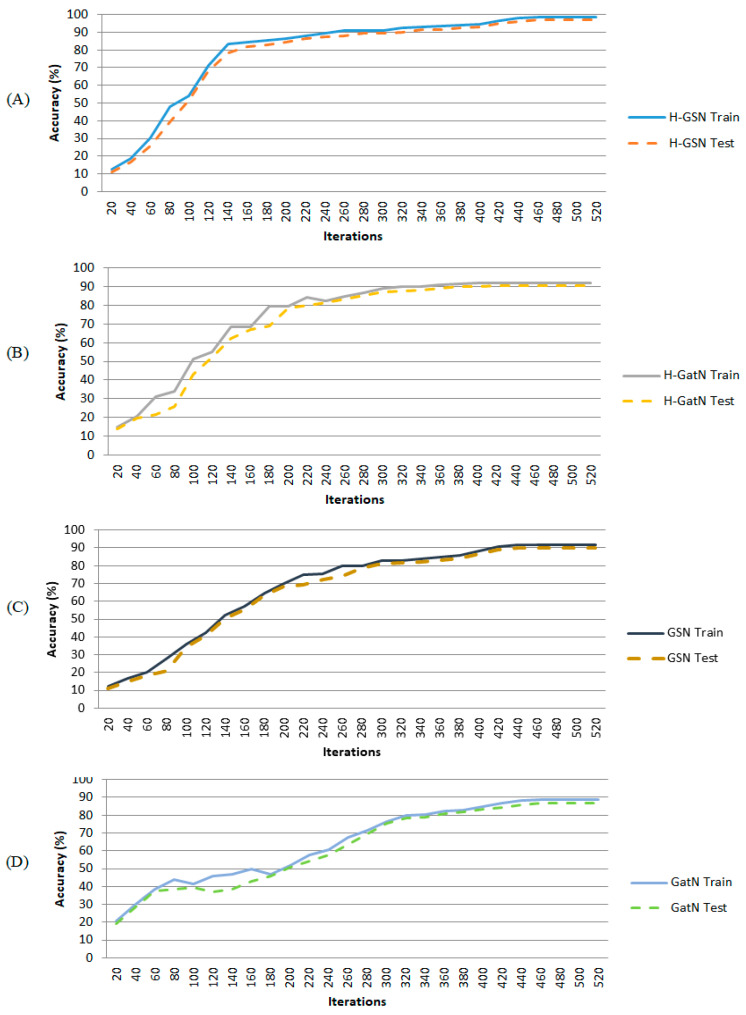
Accuracy plots for training the DataCo corresponding to the 4-category shipping mode prediction scenario ((**A**) H-GSN, (**B**) H-GatN, (**C**) GSN, (**D**) GatN).

Figure 9 shows the performance of the proposed H-GSN and H-GatN for DataCo based on the accuracy corresponding to the prediction of four different shipment modes. The graph attention is used in the H-GatN instead of the GraphSAGE layers. Corresponding to this figure, Adam optimizer with an optimal learning rate of 0.0001 and an optimum weight decay of 4 × 10^−4^ has been used, taking into consideration the cross-entropy for the first segment of the network and the total loss corresponding to the pseudo-code for the ensemble segment of the proposed network.

This figure illustrates the accuracy plots for H-GSN, H-GatN, GSN and GatN. The H-GSN is the proposed ensemble network of parallel GraphSAGE, convolutional and LSTM layers. The H-GatN is the ensemble network consisting of parallel networks of graph attention, convolutional and LSTM layers.

**Figure 10:**
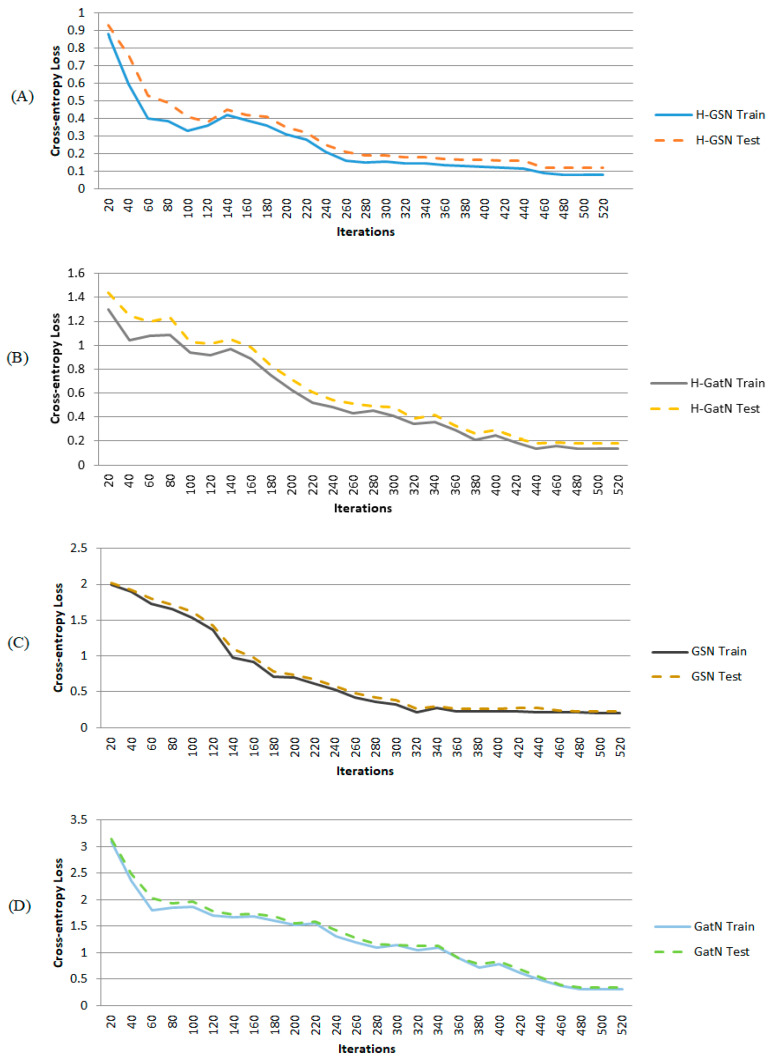
Loss plots for training the DataCo corresponding to the 4-category shipping mode classification scenario ((**A**) H-GSN, (**B**) H-GatN, (**C**) GSN, (**D**) GatN).

The GSN is our proposed GraphSAGE network without convolutional and LSTM parts, and finally, the GatN is the proposed graph attention network without convolutional and LSTM parts. Considering the same number of iterations corresponding to four different methods, the proposed hybrid GraphSAGE demonstrates better performance.The loss plots for DataCo training for the 4-class transport mode classification scenario ((A) H-GSN, (B) H-GatN, (C) GSN, (D) GatN) are shown in Figure 10. The GraphSAGE and graph attentional methods have a weak performance in comparison to the hybrid ones. Four layers of graph attention networks have been considered for H-GatN and GatN. As can be seen, more than 400 iterations have been considered for all methods utilizing a 10-fold cross-validation.

Table 14 reports the performance metrics considering the DataCo for the prediction of the delivery status and logistics shipment type for different methods. This table shows the on-time delivery and late delivery status prediction accuracy. In addition, it demonstrates the precision, F1-score and recall considering various orders for the hybrid GraphSAGE network, hybrid graph attention, non-hybrid GraphSAGE and non-hybrid graph attention methods.

**Figure 11:**
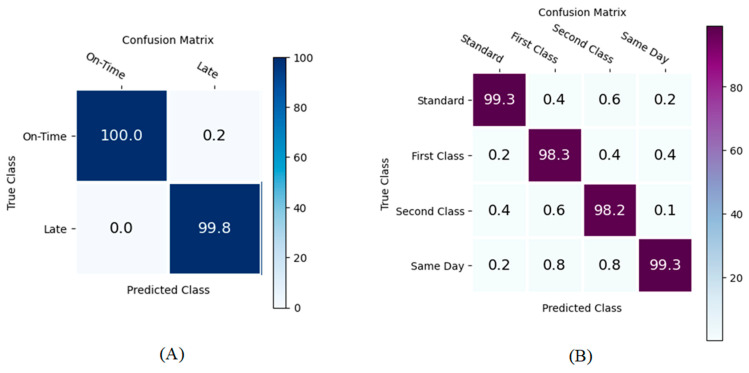
The confusion matrix for DataCo regarding (**A**) logistics delivery risk and (**B**) logistics shipping mode.

The confusion matrix is a valuable way of confirming the efficiency of the proposed method. Figure 11 delineates the confusion matrix for the DataCo dataset regarding logistics delivery status and logistics shipment mode prediction of ‘standard’, ‘first class’, ‘second class’ and ‘same day’. The performance metrics of the proposed method (accuracy, precision, recall, F1 score) on the transportation database are presented in Table 15.

**Figure 12:**
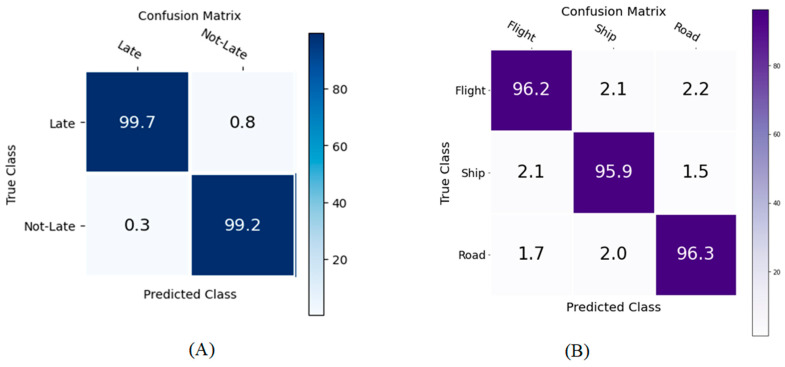
The confusion matrix for the Shipping database regarding (**A**) reached time and (**B**) mode of shipment.

**Figure 13:**
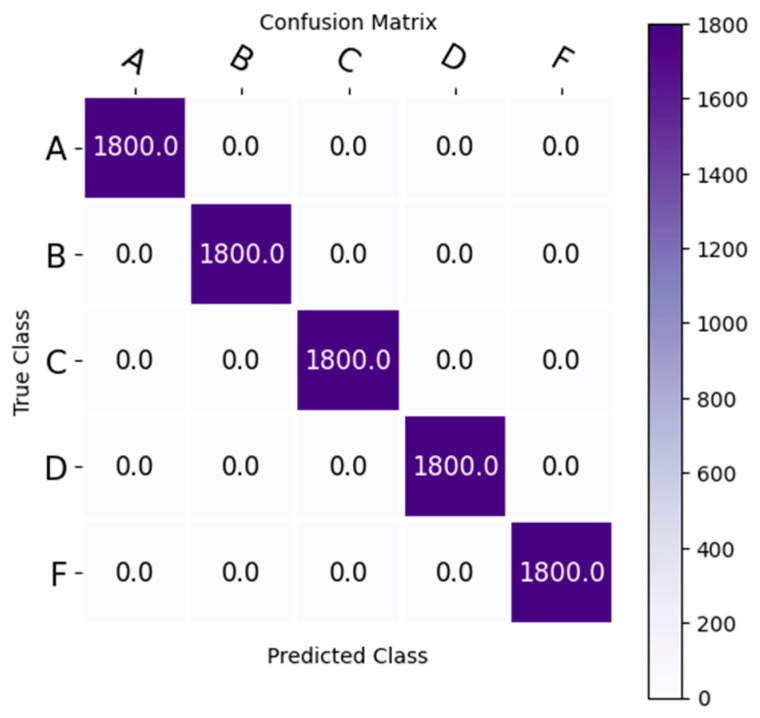
The confusion matrix regarding the logistics problem for the Shipping database regarding warehouse locations in numbers.

Figure 12 shows the performance of the proposed H-GSN considering the Shipping dataset. Figure 13 is the confusion matrix for the classification of the warehouse types considering our proposed H-GSN for the logistics parameter of warehouse location prediction according to the Shipping database.

**Figure 14:**
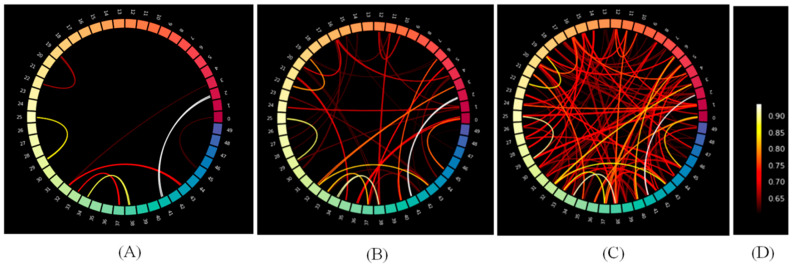
Circular connectivity of the Hospital Supply Chain dataset. (**A**) Threshold = 0.95, (**B**) threshold = 0.8, (**C**) threshold = 0.6, (**D**) color bar.

The circular connectivity is shown in Figure 14 for three sample thresholds of the Hospital Supply Chain dataset. Three threshold levels for adjacency matrix construction of 0.95, 0.8 and 0.6 are considered as the search scope during the training and cross-validation stage corresponding to the Hospital Supply Chain database.

**Figure 15:**
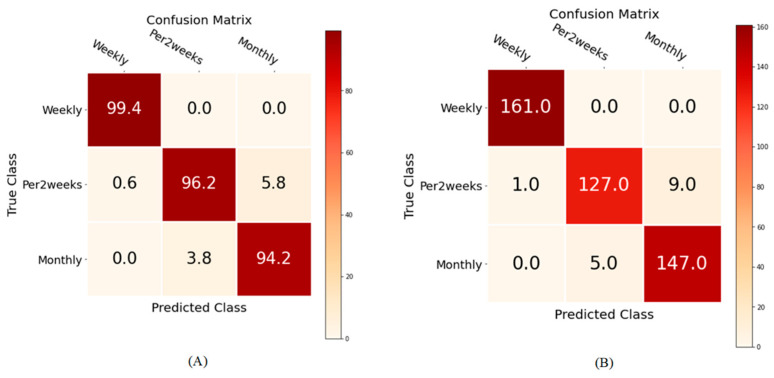
Confusion matrix for restocking lead time prediction in Hospital Supply Chain dataset; (**A**) percentage, (**B**) numeral.

**Figure 16:**
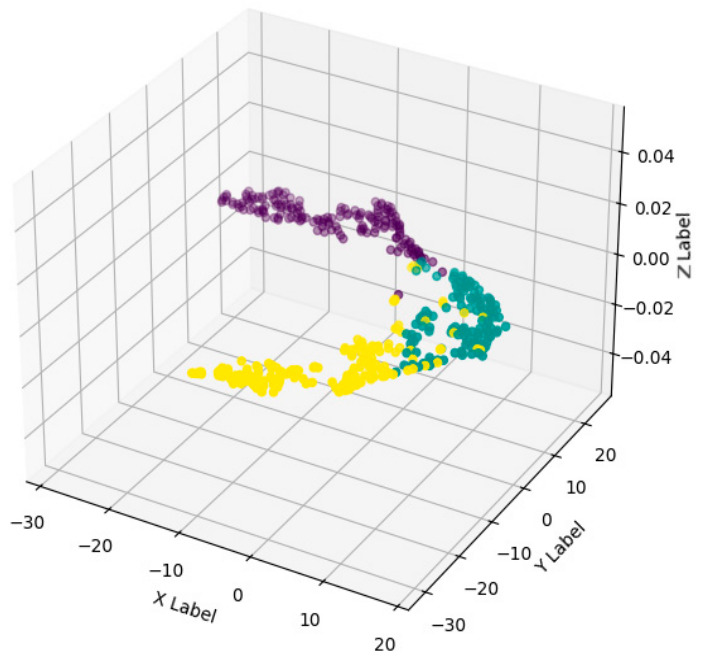
The 3-dimensional T-SNE plot for logistics prediction considering the Hospital Supply Chain database.

Figure 15 provides performance metrics for the proposed H-GSN for the lead time prediction scenario of the Hospital Supply Chain dataset. The lead time features have been separated into three categories of ‘weekly’, ‘per 2 weeks’ and ‘monthly’. The confusion matrix shows the performance of the proposed method for this 3-category logistics prediction. Figure 16 illustrates a good separation of the logistics categories considering the T-SNE plot for this type of healthcare database. It shows the efficacy of the proposed method for healthcare logistics forecasts.

**Figure 17:**
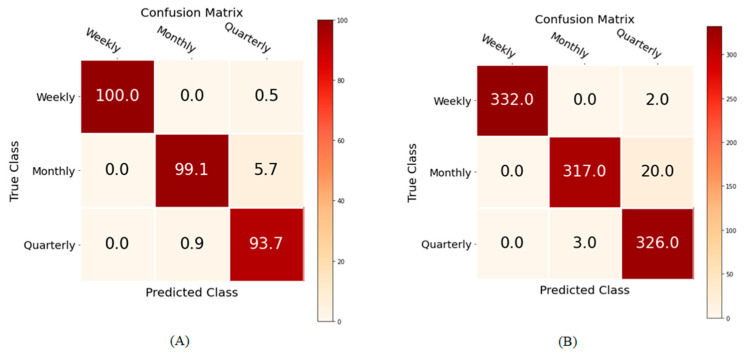
Confusion matrix for restocking lead time prediction in Pharmaceutical Supply Chain dataset; (**A**) percentage, (**B**) numeral.

Figure 17 provides performance metrics for the proposed H-GSN for the logistic restocking strategy prediction scenario of the Pharmaceutical dataset. The restocking strategy features have been separated into three categories of ‘Weekly’, ‘Monthly’ and ‘Quarterly’.

**Figure 18:**
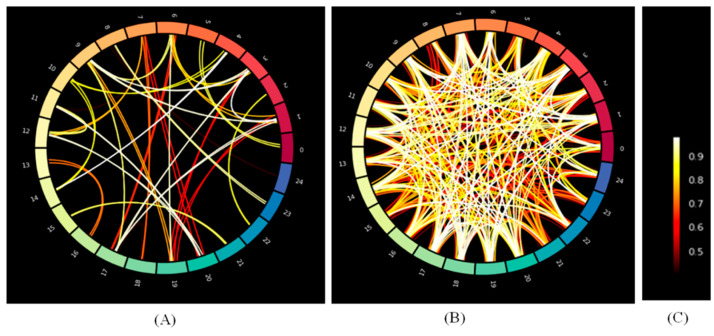
Circular connectivity of the Pharmaceutical Supply Chain dataset. (**A**) Threshold = 0.9, (**B**) threshold = 0.8, (**C**) color bar.

The circular connectivity is shown in Figure 18 for three sample thresholds of the Pharmaceutical Supply Chain dataset. Two threshold levels for adjacency matrix construction of 0.9 and 0.8 are considered as the search scope during the training and cross-validation stage, corresponding to the Pharmaceutical Supply Chain database. 

**Figure 19:**
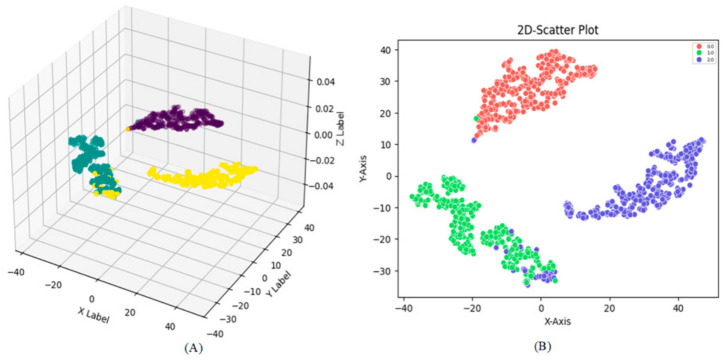
The T-SNE plots for logistics restocking lead time prediction in the Pharmaceutical dataset. (**A**) 3d (**B**) 2d.

The confusion matrix shows the performance of the proposed method for this 3-category logistics prediction for the Pharmaceutical dataset. Figure 19 illustrates a good separation of the logistics categories considering the T-SNE plot for this healthcare Pharmaceutical database. It shows the efficiency of the proposed method for healthcare logistics prediction.

**Figure 20:**
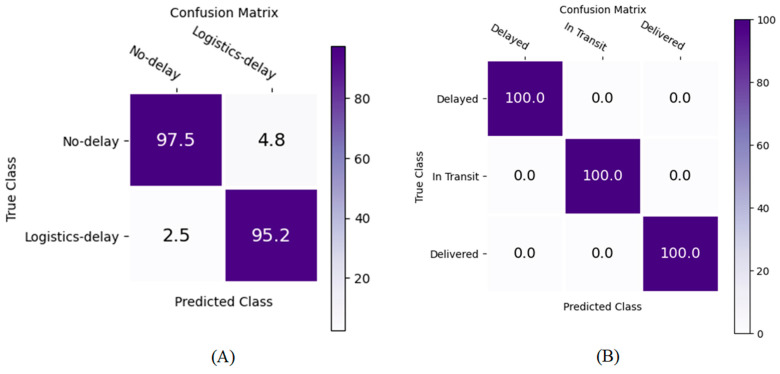
The confusion matrix for the Smart Logistics database regarding different scenarios: (**A**) logistics delay, (**B**) shipment status.

**Figure 21:**
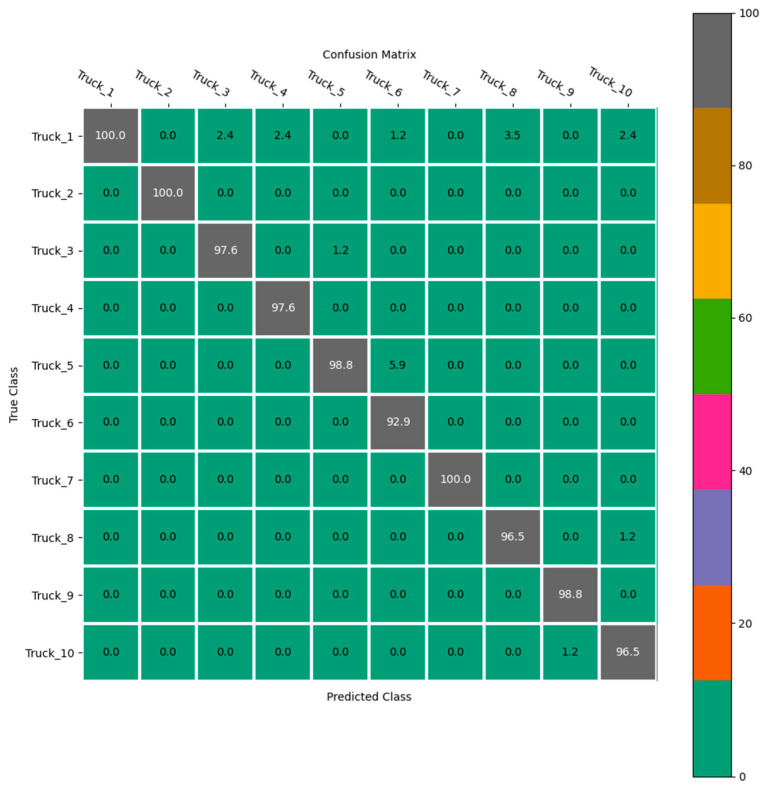
The confusion matrix for the Smart Logistics database regarding Logistics ID (Truck ID).

**Figure 22:**
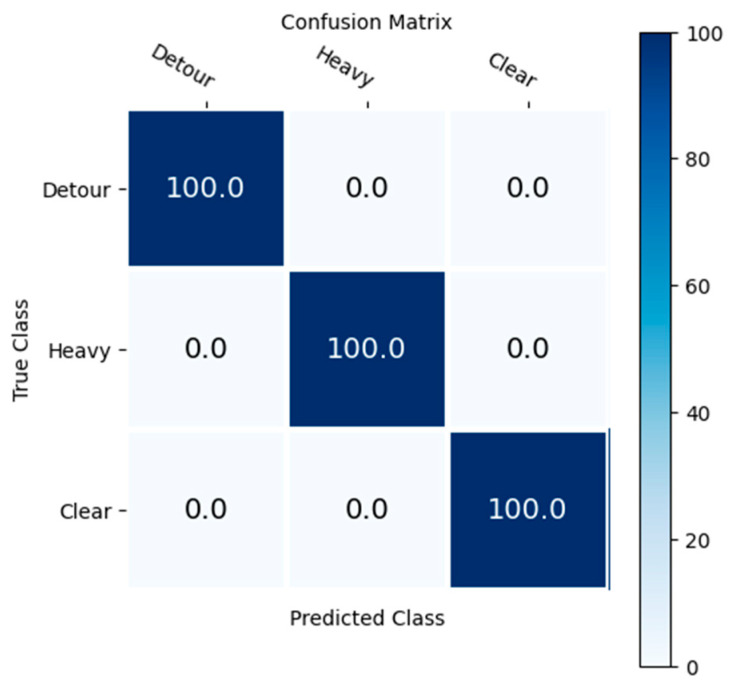
The confusion matrix for the Smart Logistic database regarding traffic status.

Table 16 provides performance metrics for the proposed H-GSN considering different logistics scenarios regarding the Smart Logistics database. Three logistics scenarios are considered for the evaluation of the efficiency of the proposed method corresponding to this benchmark dataset. The 10-category logistics ID prediction, the 3-category shipment status forecast and the prediction of logistic delay are the three scenarios considering this supply chain logistics database. The confusion matrices in Figure 20, Figure 21 and Figure 22 corresponding to each scenario of logistics delay, logistics shipment status, logistics ID and logistics traffic status confirm the efficiency of the proposed Smart Logistics supply chain model.

**Figure 23:**
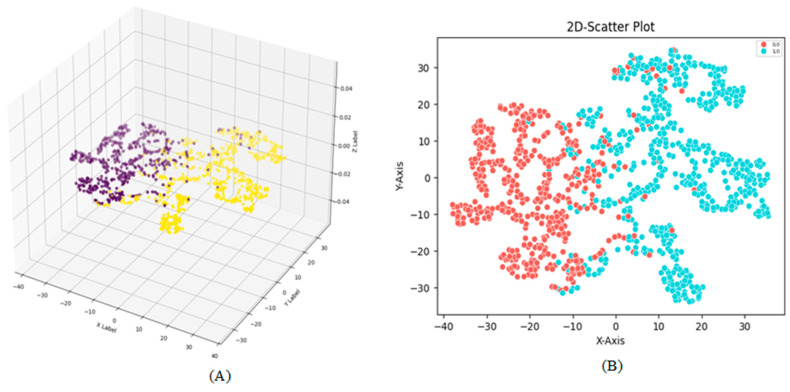
The T-SNE plot for the Smart Logistics database regarding logistics delay. (**A**) 3-dimensional and (**B**) 2-dimensional plot.

**Figure 24:**
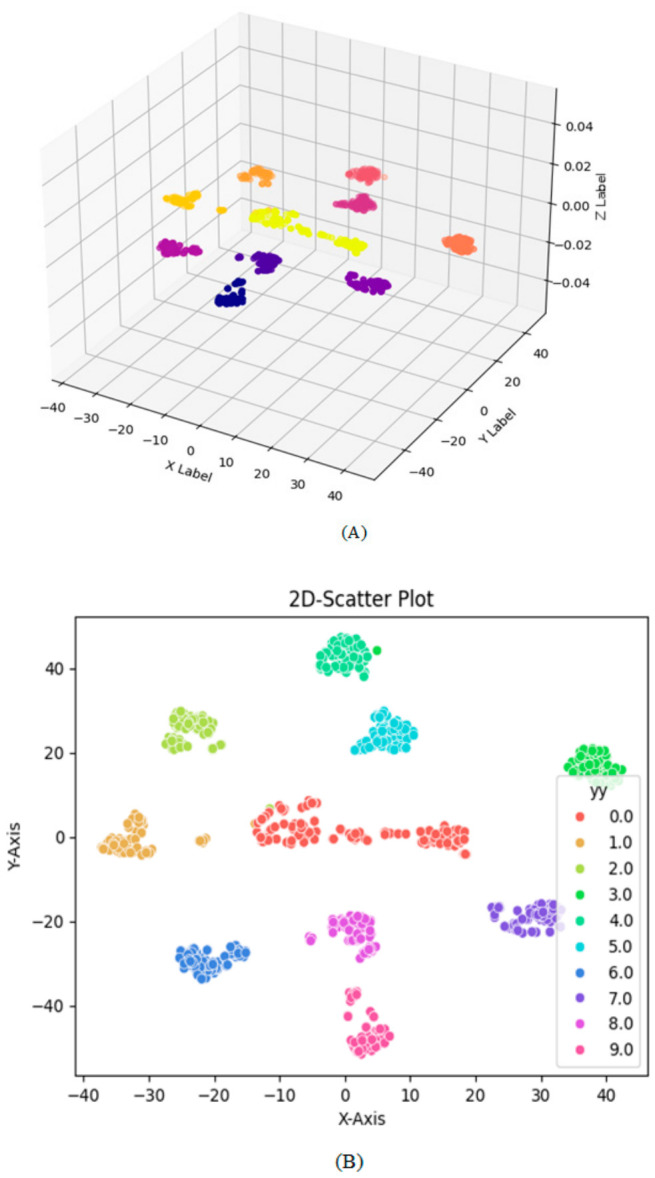
T-SNE plots for logistics ID in smart logistics. (**A**) 2-dimensional and (**B**) 3-dimensional.

**Figure 25:**
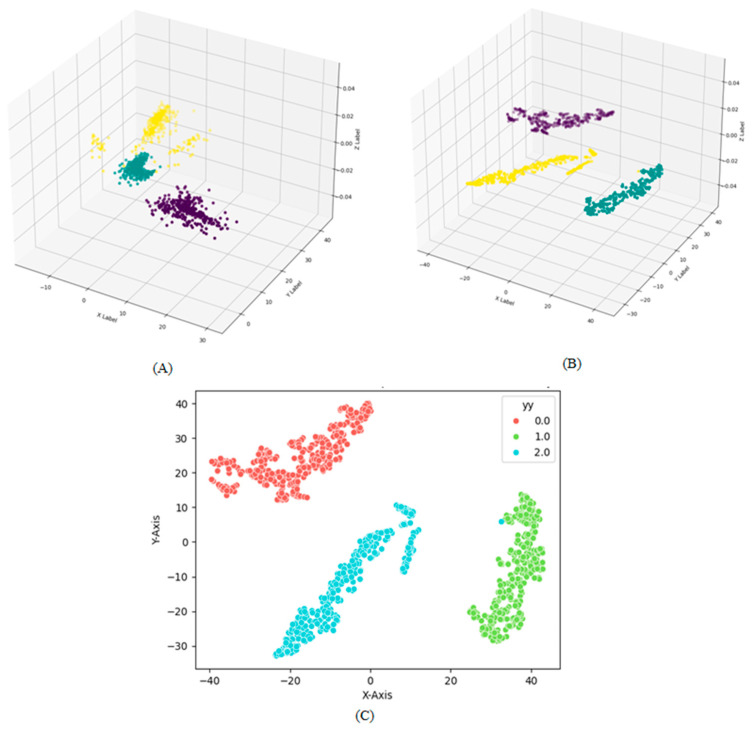
T-SNE plots for Shipment Status in Smart Logistics. (**A**) 3-dimensional output of the proposed network, (**B**) 3-dimensional after TSNE, (**C**) 2-dimensional TSNE.

Figure 23, Figure 24 and Figure 25 are tangible illustrations of the performance according to the proposed smart model for logistics prediction regarding two-category, ten-category and three-category logistics scenarios.

Each of these T-SNE plots has been provided in 2-dimensional and 3-dimensional modes to present and emphasize the efficiency of the proposed Smart Logistics model. Figure 22 is the illustration of the proposed network performance as the T-SNE plot considering the 2-category logistics delay scenario. Figure 23 and Figure 24 showcase the good performance corresponding to 10-category logistics ID and 3-category logistics traffic status prediction, respectively.

Table 17 shows the performance metrics of the proposed method in comparison to previous methods for the Smart Logistics database. As can be seen, our proposed geometric hybrid GraphSAGE network outperforms the other methods of Non-graph LSTM [53], Chebyshev convolutional-based method [39], Random Forest [54], GNN-based [55], KNN [56], XGBoost [57] and logistic regression. As can be seen, our proposed geometric hybrid network outperforms the other conventional methods. Furthermore, Table 18 illustrates the comparison with other state-of-the-art algorithms for the healthcare supply chain datasets. Also, it shows efficient performance of the proposed method in comparison to the GIN-based graph network [58] and the transformer network [59].

To investigate the effect of different parameters on the optimality of the performance, we execute an extended experiment. In order to evaluate the effect of alternating the number of sequential GraphSAGE layers, a series of training procedures is performed for different numbers of sequential GraphSAGE layers.

**Figure 26:**
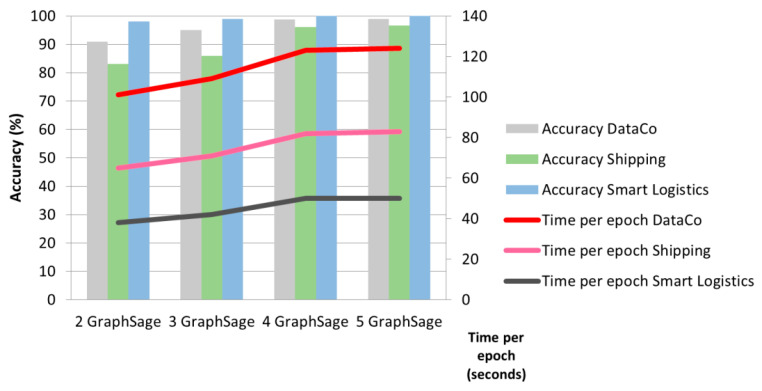
Training accuracy and time per epoch with different numbers of GraphSAGE layers for the logistics shipment mode scenario.

Figure 26 showcases the results of tuning for 2, 3, 4 and 5 sequential GraphSAGE layers. Setting the sequential layers to more than four in this case study does not improve the performance; it affects the computational complexity. This figure showcases the incremental trend of the training time per epoch during cross-validation of the proposed H-GSN.

Another dataset named the SupplyGraph has been analyzed in order to skip the graph embedding stage and consider the pre-defined edge index in this dataset to calculate the adjacency matrix. Table 19 confirms the efficiency of the proposed method regarding the SupplyGraph for predicting the 25 different logistics categories according to the manufacturing plant code. The proposed method for the plant code category prediction regarding the SupplyGraph database verifies its performance in the case of logistics parameter forecasting.

**Figure 27:**
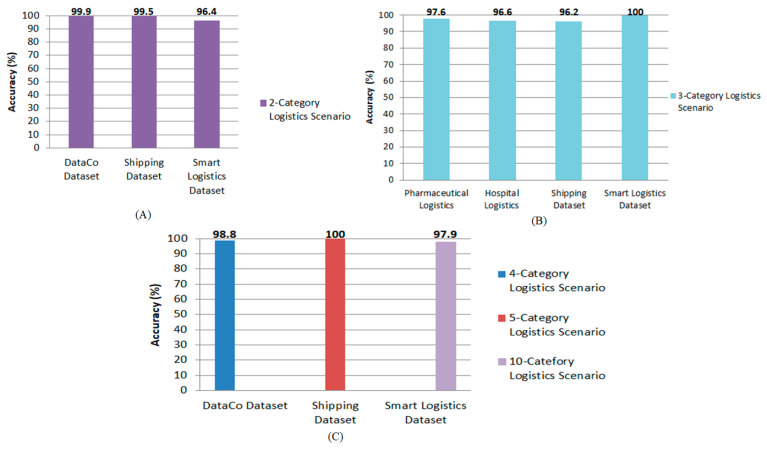
The proposed network performance considering different logistics scenarios: (**A**) 2-category, (**B**) 3-category and (**C**) 4-category, 5-category and 10-category logistics problem.

The focus of this study is on improving an intelligent supply chain logistics model for the automatic prediction of logistics parameters. The proposed Smart Logistics supply chain model has been evaluated on five different benchmark supply chain logistics datasets. Some logistics scenarios have been assessed using these datasets to emphasize the satisfactory performance of the proposed Smart Logistics model. The number of categories regarding the logistics parameter is different. The brief graphical explanation corresponding to the performance of the proposed method for these scenarios on two healthcare logistics datasets and three logistics supply chain datasets is provided in Figure 27. This figure summarizes the performance of the proposed approach considering the five benchmark datasets.

**Figure 28:**
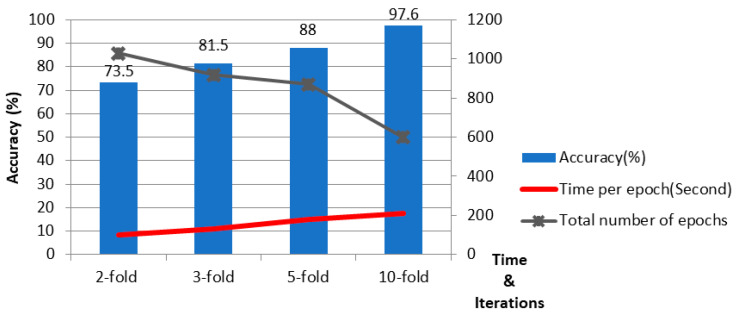
The effect of the number of folds in K-fold cross-validation regarding the Pharmaceutical Supply Chain database.

Figure 28 has been added as an outcome of evaluating the effect of considering different numbers of folds for train and test splits during cross-validation.

**Figure 29:**
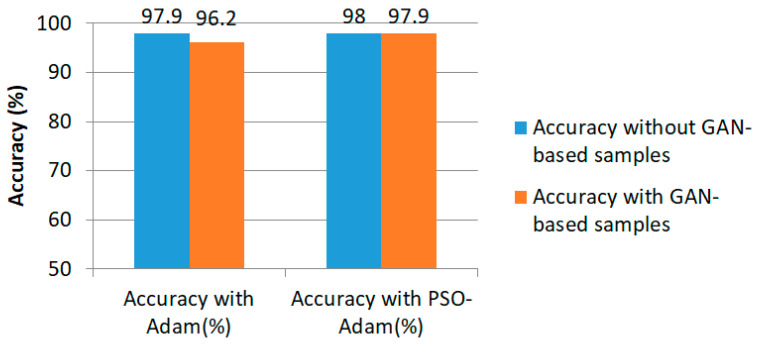
The effect of GAN-generated data on accuracy, considering a 10-category logistics ID for the Smart Logistics database.

A GAN-based strategy has been considered in order to compensate for the scarcity. A set of 1000 samples has been generated considering the architecture of the GAN according to Table 20 and Table 21 for the generator and discriminator parts, respectively. The results of the GAN-based evaluation are illustrated in Figure 29. Table 20 and Table 21 describe the details of the generator and discriminator parts of the GAN network for compensating for the data scarcity. Figure 29 emphasizes the capability of our proposed method using the PSO-Adam sequential optimization pipeline. It shows the improvement of the accuracy of the proposed method in the case of utilizing the PSO-Adam optimization. The accuracy tolerance, considering the artificial GAN-based generated samples, is acceptable regarding the Adam optimizer. The accuracy of the proposed H-GSN has been improved according to the results depicted in this figure regarding the GAN-based generated samples for augmentation.

**Figure 30:**
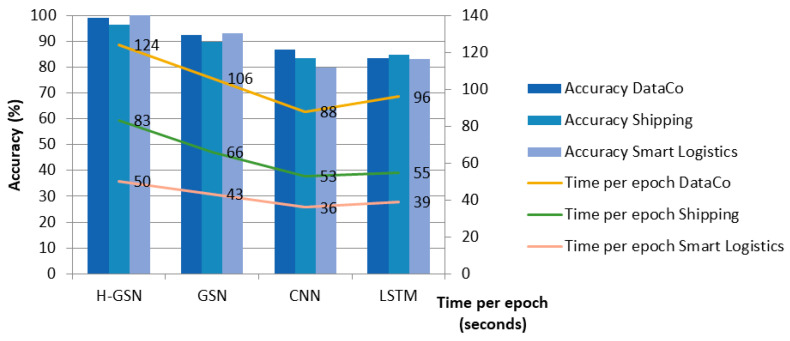
Training accuracy and time per epoch with Hybrid GraphSAGE, GrapSAGE, CNN, and LSTM architectures for the logistics shipment mode scenario.

The results of isolated training, considering each part of the hybrid network for the 3-category logistics shipment mode prediction problem, are available in Figure 30. This figure illustrates the training accuracy and the time per epoch regarding the DataCo, Shipping, and Smart Logistics datasets.

**Figure 31:**
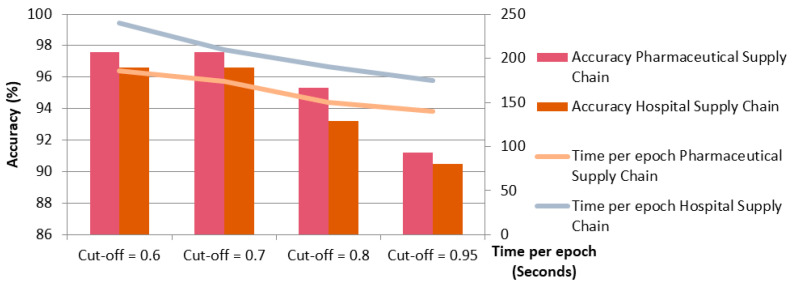
Accuracy and time per epoch with different cut-off levels for graph construction in healthcare supply chains for restocking lead time prediction.

The results of the ablation study corresponding to different cut-off levels for graph construction in Pharmaceutical and Hospital Supply Chain datasets have been explained in Figure 31.

**Figure 32:**
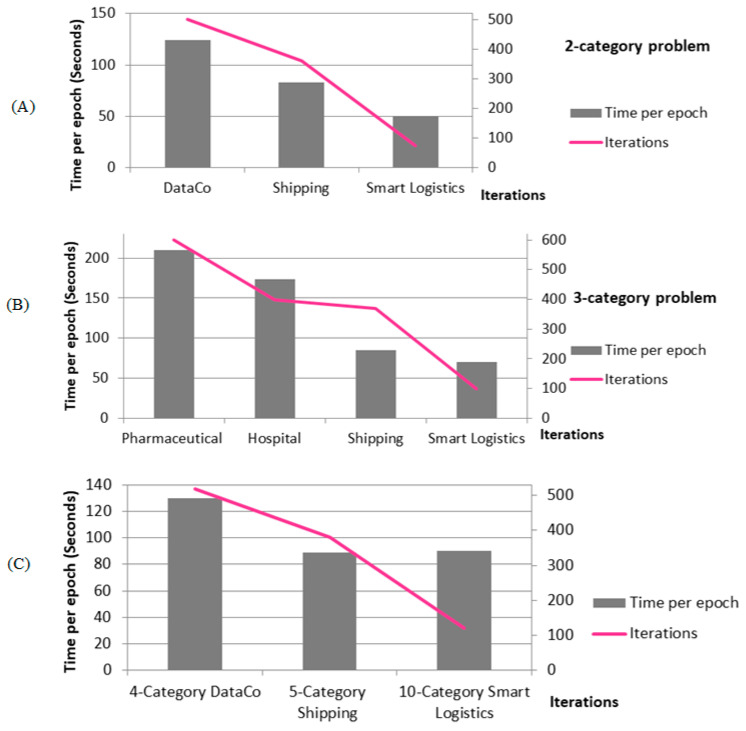
Training time per epoch and iterations for different logistics problems regarding 5 logistics datasets; (**A**) 2-category, (**B**) 3-category, (**C**) 4-category, 5-category and 10-category logistics problems.

The results of training time per epoch and the iterations specified for different logistics problems in this study are available in Figure 32.

**Figure 33:**
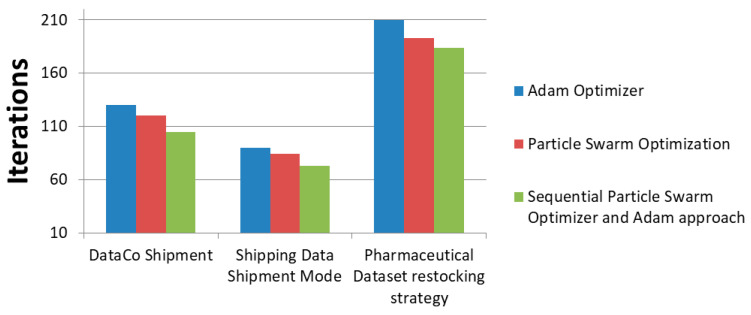
The number of iterations considering the biomimetic particle swarm optimization and the Adam optimizer.

The sequential optimizer methodology, including the biomimetic particle swarm optimizer and the Adam optimizer, has been added to boost the biomimetic aspect of the project. The individual analysis of these optimizers, along with the sequential optimizing procedure, has been performed and has been implemented. Figure 33 illustrates the effect of this biomimetic sequential approach on the iteration numbers and the increasing pace of the convergence with a lower number of iterations.

**Figure 34:**
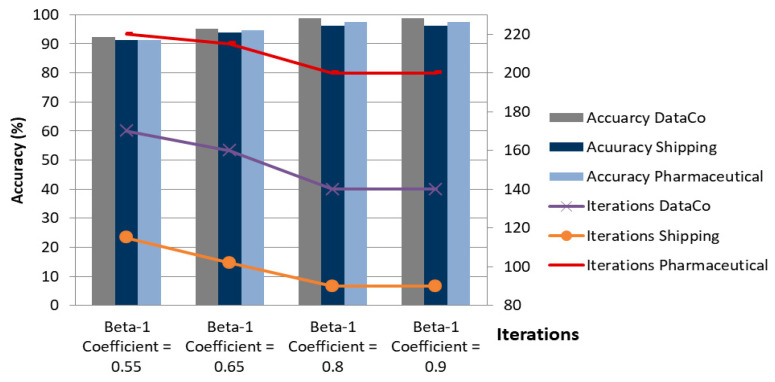
The effect of the beta coefficient in the hybrid cost function of the proposed method on the number of iterations and the accuracy regarding shipment mode prediction for the DataCo and Shipping, and the restocking strategy prediction of the Pharmaceutical dataset.

The weighted hybrid cost function in the pseudo-code is another strong biomimetic aspect of the proposed methodology, in addition to the previously mentioned aspects of our strategy. This cost function combines multiple objective metrics into a single cost using tunable weights. This is inspired by biomimetic principles (i.e., mimicking biological systems’ optimization strategies such as neural adaptation and evolutionary fitness functions). The effect of changing the beta coefficient of the weighted cost function in Algorithm 2 has been reported as a column chart in Figure 34.

We have improved an ablation study considering the biomimetic aspect of the proposed method. Figure 34 illustrates the result of this ablation study considering shipment mode prediction of the DataCo, Shipping and Pharmaceutical datasets. This figure compares the accuracy and number of iterations regarding different beta coefficients of the proposed hybrid cost function.

It illustrates that the coefficients of the non-graph parts of the proposed methodology have shown improvement in the accuracy and the number of iterations. To have a trade-off between the computational burden/complexity and the accuracy of the proposed method, the efficient amount of the beta coefficient has been obtained equal to 0.8. This figure emphasizes the results of the ablation study regarding variants of this coefficient to consider the share and coefficient for the non-graph part of the proposed hybrid GraphSAGE network.

Figure 18 illustrates the circular connectivity patterns of the Pharmaceutical Supply Chain dataset for three representative threshold values. During training and cross-validation, adjacency matrices were generated using threshold levels of 0.9 and 0.8, which were considered as the search range for model evaluation on the Pharmaceutical Supply Chain dataset. The training procedure of each fold has been performed with iterations. The iterated K-fold cross-validation strategy leads to the convergence of the network.

**Figure 35:**
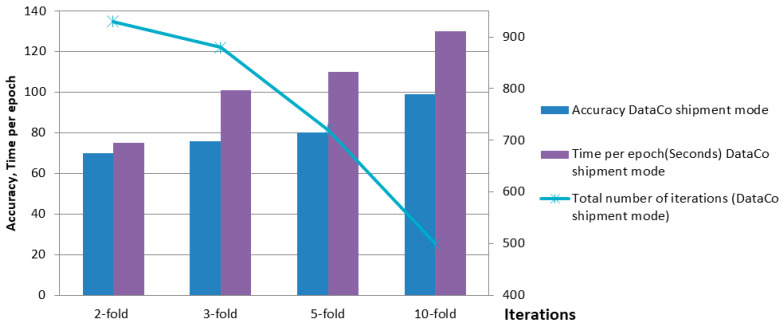
The comparison regarding different numbers of folds for K-fold cross-validation.

The results of our experiment considering different numbers of folds in cross-validation have been illustrated in Figure 35. The different number of folds in this type of cross-validation has been evaluated. Also, the other two figures have been considered for illustration of the precision and accuracy corresponding to two different prediction tasks of shipment mode of the DataCo and restocking strategy of the Pharmaceutical dataset.

**Figure 36:**
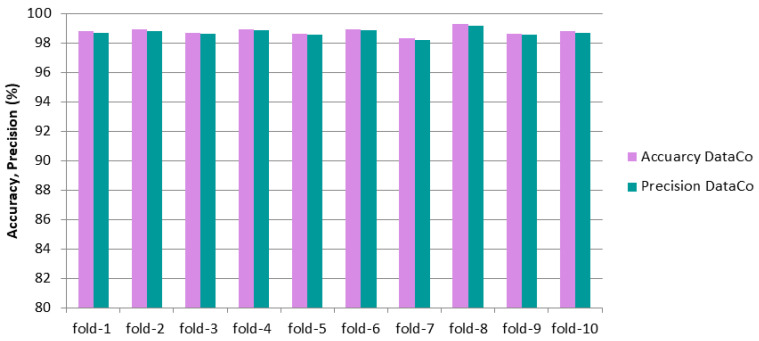
Accuracy and precision for each fold of the iterative 10-fold cross-validation for shipment mode prediction of the DataCo dataset.

**Figure 37:**
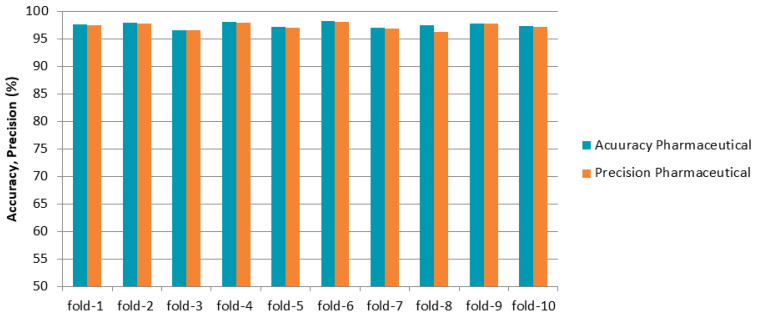
Accuracy and precision for each fold of the iterative 10-fold cross-validation for restocking strategy prediction of the Pharmaceutical dataset.

The other two figures illustrate the accuracy and precision of the proposed method corresponding to each fold during the 10-fold cross-validation procedure. Figure 36 elucidates the performance metrics regarding DataCo for shipment mode prediction. Figure 37 demonstrates the results for the restocking strategy prediction of the Pharmaceutical dataset.

In recent years, Smart Logistics and supply chain management have increasingly used optimization, AI, and graph-based methods to improve efficiency and resilience. Multi-depot routing optimizes last-mile delivery in quick commerce [60]. Blockchain improves transparency and coordination in supply chains [61]. Digital transformation enhances logistics decision-making [62]. Spatial network models support low-carbon logistics planning [63]. Urban ecosystem frameworks improve logistics sustainability [64]. Carbon–land use analysis enables green logistics optimization [65]. Urban transition models support resilient logistics design [66]. Fuzzy methods handle uncertainty in logistics allocation [67]. AI decision systems support logistics reasoning [68]. Construction models inform cost-efficient logistics optimization [69]. Multi-criteria methods improve transport safety [70]. STEAM learning supports logistics skills [71]. Peer interaction informs workforce modeling [72]. Mobility studies support routing [73]. Geotechnical models inform network stability [74]. Activity analysis supports demand estimation [75]. Ecotourism models support sustainability trade-offs [76]. Tourism studies model demand variability [77]. Personality studies inform workforce behavior [78]. Supply chain analytics optimize logistics variables [79]. Deviance theory supports anomaly detection [80]. Urban risk models support logistics planning [81]. Signal processing informs sensor analytics [82]. Economic models support demand forecasting [83]. Knowledge systems support distributed logistics learning [84]. Bayesian models support fleet prediction [85]. Structural optimization informs infrastructure design [86]. Climate models support forecasting [87]. Water models support sustainability assessment [88]. Passive design supports energy-efficient logistics [89]. Equity studies support humanitarian logistics [90]. Resilience studies inspire adaptive logistics [91]. Machine learning supports monitoring systems [92]. CFD supports process optimization [93]. Biological adaptation inspires resilient logistics [94]. Imaging supports inspection systems [95]. Meta-analyses support risk modeling [96]. Clinical studies support adaptive logistics [97]. Diagnostic fusion supports monitoring [98]. Human studies inform workforce variability [99]. Vision models support inspection [100]. Energy models support efficiency [101]. Medical AI supports intelligent logistics [102]. Biomaterials inspire resilience [103]. Bio-signaling informs optimization [104]. Neuroscience models support intelligence systems [105]. Clinical data support causal logistics analysis [106]. Biological interactions support optimization [107]. Medical anomalies support monitoring [108]. Communication models support network design [109]. Epidemic models support demand forecasting [110]. Epidemiology supports reliability modeling [111]. Disease data supports risk assessment [112]. Genetic models support high-dimensional analysis [113]. Health studies support performance modeling [114]. Infection studies support risk modeling [115]. Reviews support decision frameworks [116]. Emotion models support adaptive interfaces [117]. Data cleaning improves predictive systems [118]. IoT blockchain enables real-time logistics monitoring [119]. Accident detection improves safety systems [120]. Blockchain supports decentralized logistics [121]. Social media enables demand prediction [122]. Information systems support digital logistics [123]. Reinforcement learning enables adaptive allocation [124]. Intrusion detection improves cybersecurity [125]. Machine learning supports optimization [126]. FinTech supports payments [127]. AI behavior models support demand prediction [128]. Trust in AI affects automation [129]. Vision models support perception [130]. Ranking methods support optimization [131]. Clustering supports analytics [132]. Ontologies support semantics [133]. Blockchain enables automation [134]. Declarative models support control [135]. SHACL ensures validation [136]. Smart assets support digital twins [137]. Multi-objective optimization improves design [138]. Network models support risk analysis [139]. Fuel models support elasticity [140]. Equity studies support fairness [141]. Behavior models support sustainability [142]. Financial uncertainty supports risk analysis [143]. Workforce studies support labor risk [144]. Driving models support fleet intelligence [145]. Ecosystem networks inspire optimization [146]. Ecosystem planning frameworks support resilient logistics system design [147]. Finally, multidisciplinary Smart Logistics planning frameworks integrate sustainability, resilience, and AI-driven optimization in complex urban logistics systems [148].

There are some limitations regarding our proposed methods. In the following, we refer to some of these restrictions to emphasize and assess in our future work on supply chain management. A multi-objective optimization in supply chain logistics networks plays an important role in constructing resilient and efficient management. It is necessary to consider this type of optimization in future work. In this case, the training can be performed considering multiple target variables at the same time, and multi-objective optimization can be developed in future works to introduce a deep end-to-end network for simultaneous management of supply chain sustainability and supply chain logistics parameters.

Another important factor is considering a knowledge graph learning method to acquire a heterogeneous graph of supply chain datasets. In this study, a one-type connection of the nodes has been considered to construct a homogeneous graph. It would be practical to obtain a heterogeneous graph and impose it on a deep network architecture.

These limitations will be considered in future work about supply chains and supply chain logistics management to introduce innovations for supply chain management.

## 6. Conclusions

In this paper, a novel architecture of a brain-inspired hybrid geometric deep network is proposed to provide an intelligent supply chain logistics model. This Smart Logistics model solves the problem of logistics and logistics risk management in a supply chain. In addition, it is a deep, intelligent model proposed to improve the resiliency and sustainability of a supply chain. The proposed model architecture is used for automatic logistics management regarding the DataCo, Shipping and Smart Logistics databases and two healthcare supply chain logistics datasets.

The optimizer in the proposed methodology performs sequential optimization using the biomimetic particle swarm optimizer and the Adam approach (PSO-Adam), considering the hybrid cost function. The main challenges in this paper are utilizing graph theory along with deep network architectures and considering the connectivity between nodes to extract the hidden states of supply chain principal vectors. This geometric graph connectivity is inspired by the functional connectivity between different brain lobes during neural interactions. The brain-inspired hybrid strategy is a novel method for logistics automation and multi-task supply chain logistics management. It is a multi-task network that facilitates logistics management in a supply chain, along with strengthening the sustainability and risk management regarding logistics in a supply chain. The efficiency of the proposed method for creating a Smart Logistics model is explored on five supply chain logistics datasets. The proposed H-GSN provides a transparent and resilient multi-task logistics supply chain model. Furthermore, it is a cost-efficient logistics model with consideration of the prediction capabilities. There are ten logistics problems, and the obtained evaluation metrics emphasize the efficiency of the proposed method in the prediction of the logistics parameters, preventing some logistics risks, helping to increase the productivity of the supply chain, and leading to improved customer satisfaction. Also, the logistics efficiency affects the business revenue growth, internal processes, and operations in a business and positively enhances and boosts the industry reputation.

## Figures and Tables

**Table 1:** Summary of transportation and logistics techniques used in supply chain management.

Reference	Method	Contribution	Advantages	Disadvantages
Akbari-Aghghale et al. [44]	Closed-loop poultry supply chain	Poultry supply chain based on a meta-heuristic approach	Multi-task approach, minimizing the supply chain cost	-
Jang et al. [31] 2024	Stochastic programming	Hydrogen supply chain	Execute the sample average approximation	Computational burden is not efficient
Niu et al. [23] 2024	New design for the supply chain network	Including location choices for manufacturing plants	Efficient distribution	Accuracy is not efficient
Zulqarnain et al. [34] 2024	Fuzzy approach	Utilizing aggregation	Efficient management	Accuracy not improved
Sesar et al. [21] 2023	Anomaly at unfolding	Execute unfolding	Waste management	Sensitivity not improved
Tsolaki et al. [45] 2023	Machine Learning methodologies	Assessing transportation parameters	Reducing arrival time	Accuracy not improved
Deng et al. [46] 2023	Robust optimization approach	Identifying challenges	Handling transport services	F1-score not improved
Matenga et al. [33] 2022	Industrial development operations	Executing customer management process	Efficient management	Computational burden not improved
Anwer et al. [35] 2022	Quantitative approach	Considering deficiencies in transportation	Analyzing the primary data	No improvement in minimizing the complexity
Fartaj et al. [47] 2020	Disturbance factors in logistics	Computing the interrelationship in supply chain logistics	Efficient management	No improvement in error reduction

**Table 2:** DataCo specifications.

Feature Number	Feature	Format	Feature Number	Feature	Format
**1**	Type	Debit-0, Transfer-1, Payment-2, Cash-3	**7**	Longitude of location	Numeral
**2**	Real days of shipping	Digit	**8**	Discount	Numeral
**3**	Planned days of shipment	Digit	**9**	Discount rate	Numeral
**4**	Gain for customer order	Numeral	**10**	Total or-der	Numeral
**5**	Sales for consumer	Numeral	**11**	Rate of order profit	Numeral
**6**	Latitude of location	Numeral	**12**	Order state	8 different text

**Table 3:** Target tasks for DataCo.

Feature Number	Target Feature	Explanation	Number of Data Samples for Each Target Category
**1**	Delivery status	1-On-time, 2-Late delivery	18,000
**2**	Shipping mode	1-Standard Class, 2-First Class, 3-Second Class, 4- Same Day	9000

**Table 4:** Shipping dataset features.

Shipping	Feature	Explanation	Shipping	Feature	Explanation
**1**	Customer care calls	Digit	**5**	Product importance	3 types of word (low, medium, high)
**2**	Customer rating	Digit	**6**	Gender	2 types of word (F,M)
**3**	Cost of the product	Numeral	**7**	Discount offered	Numeral
**4**	Prior purchases	Digit	**8**	Weight in grams	Numeral

**Table 5:** Target tasks in the Shipping dataset.

Shipping	Target Feature	Explanation	Number of Data Samples for Each Target Category
**1**	Warehouse	5 categories (A, B, C, D, F)	1056
**2**	Mode of Shipment	3 categories (Flight, Ship, Road)	1760
**3**	Reached On-Time	2 categories (Late, On-Time)	2640

**Table 6:** Smart Logistics dataset features.

Smart Logistics	Feature	Explanation	Smart Logistics	Feature	Explanation
**1**	Latitude	Numeral	**6**	Humidity	Numeral
**2**	Longitude	Numeral	**7**	Traffic Status	3 categories (Detour, Heavy, Clear)
**3**	Inventory_Level	Numeral	**8**	Waiting Time	Numeral
**4**	Shipment_Status	3 categories (Delayed, In Transit, Delivered)	**9**	User Transaction Amount	Numeral
**5**	Temperature	Numeral	**10**	User Purchase Frequency	Digit

**Table 7:** Target tasks in the Smart Logistics dataset.

Smart Logistic	Target Feature	Explanation	Number of Data Samples for Each Target Category
**1**	Truck_ID	10 categories	100
**2**	Shipment Status	3 categories (Delayed, In Transit, Delivered)	300
**3**	Traffic Status	3 categories (Detour, Heavy, Clear)	300
**4**	Logistics Delay	2 categories (Late, On-Time)	500

**Table 8:** Features of the Hospital Supply Chain dataset.

Hospital Supply Chain Dataset	Feature	Explanation	Feature Type
**1**	Current Stock	Numeral	Input
**2**	Min Required	Numeral	Input
**3**	Max Capacity	Numeral	Input
**4**	Unit Cost	Numeral	Input
**5**	Average Usage Per Day	Numeral	Input
**6**	Vendor ID	3 Categories (V001, V002, V003)	Input
**7**	Item Type	5 Categories (Ventilator, Surgical Machine, IV Drip, X-ray Machine, Gloves)	Input
**8**	Restock Lead Time	3 Categories	Target

**Table 9:** Features of the Pharmaceutical Supply Chain dataset.

Pharmaceutical Supply Chain Dataset	Feature	Explanation	Feature Type
**1**	Drug Name	4 Categories (Metformin, Lisinopril, Insulin, Atrovastatin)	Input
**2**	Demand Forecast	Numeral	Input
**3**	Optimal Stock Level	Numeral	Input
**4**	Restocking Strategy	3 Categories (Weekly, Monthly, Quarterly)	Target

**Table 10:** Layers of the GraphSAGE of the proposed H-GSN method.

Dataset	Layer	Layer Name	Activation Function	Dimension of Weight Array	Dimension of Bias	Number of Parameters
**Shipping for Shipment Mode**	1	GraphSAGE	Relu	[1, 8, 8]	[8]	72
	3	Batch normalization	-	[8]	[8]	16
	4	GraphSAGE	Relu	[1, 8, 5]	[5]	45
	6	Batch normalization	-	[5]	[5]	10
	7	GraphSAGE	Relu	[1, 5, 3]	[3]	18
	8	Batch normalization	-	[3]	[3]	6
	9	GraphSAGE	Relu	[1, 3, 3]	[3]	12
	10	Batch normalization	-	[3]	[3]	6
**Smart Logistics for Logistics ID**	1	GraphSAGE	Relu	[1, 10, 10]	[10]	110
	2	Batch normalization	-	[10]	[10]	20
	3	GraphSAGE	Relu	[1, 10, 10]	[10]	110
	4	Batch normalization	-	[10]	[10]	20
	5	GraphSAGE	Relu	[1, 10, 10]	[20]	110
	6	Batch normalization	-	[10]	[10]	20
	7	GraphSAGE	Relu	[1, 10, 10]	[10]	110
	8	Batch normalization	-	[10]	[10]	10
	9	GraphSAGE	Relu	[1, 10, 10]	[10]	110
	10	Batch normalization	-	[10]	[10]	20

**Table 11:** Details of the LSTM part of the proposed method.

Data	Layer	Layer Name	Number of Layers
Shipping (Logistic ID)	1	LSTM	5
	2	Linear	1
Smart Logistics (Shipment Mode)	1	LSTM	5
	2	Linear	1

**Table 12:** Details of the convolutional part of the proposed method.

Data	Layer	Layer Name	Activation Function	Output Dimension	Stride Shape	Size of Window	Number of Kernels	Number of Weights
Shipping (Logistic ID)	1	Convolution 1-D	LeakyReLU (alpha = 0.1)	(10, 10, 5)	1 × 1	1 × 5	10	510
	2	Convolution 1-D	LeakyReLU (alpha = 0.1)	(10, 10, 5)	1 × 1	1 × 5	10	502
Smart Logistics (Shipment Mode)	1	Convolution 1-D	LeakyReLU (alpha = 0.1)	(8, 8, 5)	1 × 1	1 × 5	8	328
	2	Convolution 1-D	LeakyReLU (alpha = 0.1)	(3, 8, 5)	1 × 1	1 × 5	3	123

**Table 13:** Search space and optimal values for hyperparameters of the proposed method.

Parameters	Search Space	Optimum Value
Optimizer of GraphSAGE	Adam, SGD	Adam
Cost function of GraphSAGE	MSE, Cross-Entropy	Cross-Entropy
Number of Sage layers	2, 3, 4	4
Learning rate of GraphSAGE	0.1, 0.01, 0.001	0.001
Window size	10, 20, 30	20
Optimizer of convolution and LSTM	Adam, SGD	Adam
Learning rate of convolution and LSTM	0.01, 0.001, 0.0001, 0.00001	0.0001

**Table 14:** Performance metrics of the proposed method (Accuracy, Precision, Recall, F1-score) regarding DataCo.

Logistics Delay Categories of the DataCo Dataset	H-GSN	GSN	H-GatN	GatN	Shipping Mode Categories of the DataCo Dataset	H-GSN	GSN	H-GatN	GatN
**Overall accuracy**	99.9 ± 0.5	92.56 ± 1.1	94.98 ± 0.6	90.45 ± 1.03	**Overall accuracy**	98.7 ± 0.2	91.82 ± 2.26	92.15 ± 1.98	88.65 ± 2.58
**Precision**	99.9 ± 0.5	92.5 ± 1.3	94.9 ± 0.7	90.74 ± 1.22	**Precision**	98.7 ± 0.3	91.8 ± 2.3	91.1 ± 1.5	86.6 ± 2.6
**F1-score**	98.9 ± 0.3	92.5 ± 0.9	94.9 ± 0.5	90.42 ± 1.35	**F1-score**	98.7 ± 0.4	91.8 ± 2.4	92.1 ± 1.4	86.6 ± 2.7
**Recall**	99.9 ± 0.4	92.5 ± 0.9	94.9 ± 0.4	90.08 ± 1.26	**Recall**	98.7 ± 0.2	91.8 ± 2.3	91.1 ± 1.5	86.6 ± 2.3

**Table 15:** Performance Metrics of the proposed method (Accuracy, Precision, Recall, F1-score) in the Shipping database.

Logistics Reached Time of Shipping Database	H-GSN	H-GatN	Logistics Mode of Shipment of Shipping Database	H-GSN	H-GatN	Logistics Warehouse location of Shipping Database	H-GSN	H-GatN
**Overall accuracy**	99.4 ± 0.1	96.54 ± 0.55	**Overall accuracy**	96.13 ± 0.21	85.46 ± 2.34	**Overall accuracy**	100 ± 0	97.28 ± 0.84
**Precision**	99.4 ± 0.7	96.18 ± 1.01	**Precision**	96.1 ± 0.32	85.4 ± 2.8	**Precision**	100 ± 0	97.2 ± 0.7
**F1-score**	99.4 ± 0.9	96.03 ± 1.05	**F1-score**	96.1 ± 0.87	85.4 ± 2.7	**F1-score**	100 ± 0	97.2 ± 0.6
**Recall**	99.4 ± 0.5	96.19 ± 1.42	**Recall**	96.1 ± 0.65	85.4 ± 2.3	**Recall**	100 ± 0	97.2 ± 0.7

**Table 16:** Accuracy for multi-task prediction of the Smart Logistics database.

Smart Logistics (Logistics ID)	H-GSN	H-GatN	Smart Logistics (Shipment Status)	H-GSN	H-GatN	Smart Logistics (Logistics Delay)	H-GSN	H-GatN
**Overall accuracy**	97.8 ± 0.12	88.46 ± 1.31	**Overall accuracy**	100 ± 0	90.4 ± 2.8	**Overall accuracy**	96.35 ± 0.12	80.2 ± 1.4
**Precision**	97.8 ± 0.18	87.18 ± 1.02	**Precision**	100 ± 0	89.3 ± 1.9	**Precision**	96.38 ± 0.23	80.2 ± 1.1
**F1-score**	97.8 ± 0.21	87.03 ± 1.42	**F1-score**	100 ± 0	89.2 ± 2.5	**F1-score**	96.32 ± 0.47	80.2 ± 1.3
**Recall**	97.8 ± 0.34	87.19 ± 0.98	**Recall**	100 ± 0	89.26 ± 2.35	**Recall**	96.35 ± 0.79	80.2 ± 0.98

**Table 17:** Comparison with other state-of-the-art and conventional methods.

Method	Logistic ID Smart Logistics Database	Shipment Status Smart Logistics Database	Logistic Delay Smart Logistics	Traffic Status Smart Logistics Database
H-GSN	97.9	100	96.35	100
GIN-based graph network [58]	94.7	94.7	93.95	94.21
Non-graph LSTM [53]	94.5	94.5	93.51	94.23
Chebyshev convolutional-based method [39]	94.98	95.24	95.64	95.12
Transformer network [59]	91.4	91.4	92.1	92.1
Random Forest [54]	90.50	90.10	87.56	89.34
GNN-based [55]	81.23	92.36	90.43	91.82
BiLSTM + SVM	79.94	80.23	78.35	80.45
KNN [56]	63.44	78.23	62.64	76.43
Logistic regression	66.67	68.32	63.54	68.21
XGBoost [57]	62.42	74.06	61.13	73.15

**Table 18:** Comparison with other state-of-the-art and conventional methods for healthcare datasets.

Method	Pharmaceutical Supply Chain	Hospital Supply Chain
H-GSN	96.5	96.6
GIN-based graph network [58]	94.7	94.6
Non-graph LSTM [53]	94.3	94.3
Chebyshev convolutional-based method [39]	94.98	94.52
Transformer network [59]	91.3	91.2
Random Forest [54]	90.50	89.30
GNN-based [55]	81.23	80.40
BiLSTM + SVM	80.54	80.32
KNN [56]	63.44	62.37
Logistic regression	66.67	65.37
XGBoost [57]	62.42	60.89

**Table 19:** Accuracy for logistics plant relation classification considering the SupplyGraph database.

SupplyGraph (With Pre-Defined Edges in Dataset)	H-GSN	H-GatN	GSN	GatN
Product category in nodes (5 product codes)	100	95.32	86.71	84.18
Product category relation in edges (4 product codes)	98.8	89.43	83.88	83.23
Manufacturing plant relation in edges (25 logistics category corresponding to plant codes)	96.2	85.32	84.66	82.92

**Table 20:** The generator layers for augmentation.

Layer Type	Activation Function	Output Shape	Kernel Dimension	Stride Size	Padding	Number of Filters
Fully Connected		(5,50,8)				
Reshape layer		(5,50,8)				
1st 2-D Transposed Conv	Leaky Relu (coeff = 0.1)	(5,50,8)	1*4	1*1	yes/same	8
2nd 2-D Transposed Conv	Leaky Relu (coeff = 0.1)	(10,100,8)	1*4	2*2	yes/same	8

**Table 21:** The discriminator layers for augmentation.

Layer Type	Activation Function	Output Shape	Kernel Dimension	Stride Size	Padding	Number of Kernels
1st 2-D Conv	Leaky Relu (coeff = 0.1)	(1,5,50,4)	1*4	2*2	yes/same	4
Dropout layer (0.3)		(1,5,50,4)				
2nd 2-D Conv	Leaky Relu (coeff = 0.1)	(1,5,50,4)	1*4	1*1	yes/same	4
Dropout layer (0.3)		(1,5,50,4)				
Flatten		(1,1000)				
Dense Layer		(1,500)				
Dense Layer		(1,1)				

## Data Availability

The datasets used in this study are publicly available at the following address links: https://www.kaggle.com/datasets/vanpatangan/hospital-supply-chain; https://www.kaggle.com/datasets/mohammedashraf000/pharmaceutical-supply-chain-optimization; https://www.kaggle.com/datasets/nayanack/shipping; https://www.kaggle.com/datasets/ziya07/smart-logistics-supply-chain-dataset; https://www.kaggle.com/datasets/shashwatwork/dataco-smart-supply-chain-for-big-data-analysis; https://www.kaggle.com/datasets/azminetoushikwasi/supplygraph-supply-chain-planning-using-gnns (all accessed on 18 May 2026).

## References

[1] Sirina N., Zubkov V. (2021). Transport services management on transport and logistic methods. Transp. Res. Procedia.

[2] Xu X. (2025). Logistics distribution path optimization based on deep reinforcement learning. Procedia Comput. Sci..

[3] Ren J., Xia F. (2024). Brain-inspired artificial intelligence: A comprehensive review. arXiv.

[4] Batbaatar E., Ryu K.H. (2025). Bio-Inspired Generative Network with Knowledge Integration. Appl. Sci..

[5] Song X., Wang Y., Liu B., Liu W. (2022). Brain-inspired Hierarchical Attention Recurrent CNN for Image Classification. Proceedings of the 2022 16th IEEE International Conference on Signal Processing (ICSP), Beijing, China, 21–24 October 2022.

[6] Bertoni F., Citti G., Sarti A. (2022). LGN-CNN: A biologically inspired CNN architecture. Neural Netw..

[7] Bo L., Xu J. (2025). Logistics optimization based on biomechanical principles and bionic algorithms and its innovative approach to intelligent supply chain management. Mol. Cell. Biomech..

[8] Dragomirescu C.-G., Iliescu V. (2024). Implementation of Methods Aimed at Sustainable Development Through Bionics. Sci. Pap. Ser. E Land Reclam. Earth Obs. Surv. Environ. Eng..

[9] Culot G., Podrecca M., Nassimbeni G. (2024). Artificial intelligence in supply chain management: A systematic literature review of empirical studies and research directions. Comput. Ind..

[10] Ghazinoory S., Aghaei P. (2024). Key success factors for stability of asymmetric technological collaborations: A bionic engineering approach. J. Bus. Ind. Mark..

[11] Cao W., Yan Z., He Z., He Z. (2020). A comprehensive survey on geometric deep learning. IEEE Access.

[12] Burkhart J.G., Wu G., Song X., Raimondi F., McWeeney S., Wong M.H., Deng Y. (2023). Biology-inspired graph neural network encodes reactome and reveals biochemical reactions of disease. Patterns.

[13] da Silva A.M.B., Ferreira N.C.d.S., Braga L.A.M., Mota F.B., Maricato V., Alves L.A. (2024). Graph neural networks: A bibliometric mapping of the research landscape and applications. Information.

[14] Khedr A.M. (2024). Enhancing supply chain management with deep learning and machine learning techniques: A review. J. Open Innov. Technol. Mark. Complex..

[15] Toorajipour R., Sohrabpour V., Nazarpour A., Oghazi P., Fischl M. (2021). Artificial intelligence in supply chain management: A systematic literature review. J. Bus. Res..

[16] Raj A., Mukherjee A.A., de Sousa Jabbour A.B.L., Srivastava S.K. (2022). Supply chain management during and post-COVID-19 pandemic: Mitigation strategies and practical lessons learned. J. Bus. Res..

[17] Shidpour H., Shidpour M., Tirkolaee E.B. (2023). A multi-phase decision-making approach for supplier selection and order allocation with corporate social responsibility. Appl. Soft Comput..

[18] Pereira E.L., Morreira M.Â.L., Gomes C.F.S., dos Santos M., de Araújo Costa A.P., Chagas S.D.S.S., de Araújo Costa I.P., Kojima E.H. (2023). Supply Chain Management (SCM): An Analysis based on the CRITIC-GRA-3N Method in the selection of auto parts suppliers for an auto parts dealer in the city of Guaratinguetá. Procedia Comput. Sci..

[19] Ali M.R., Nipu S.M.A., Khan S.A. (2023). A decision support system for classifying supplier selection criteria using machine learning and random forest approach. Decis. Anal. J..

[20] Yazdani M., Torkayesh A.E., Stević Ž., Chatterjee P., Ahari S.A., Hernandez V.D. (2021). An interval valued neutrosophic decision-making structure for sustainable supplier selection. Expert Syst. Appl..

[21] Drljača M., Sesar V. (2023). Supply chain transportation management. Transp. Res. Procedia.

[22] Fu C., Liu Y.-Q., Shan M. (2023). Drivers of low-carbon practices in green supply chain management in construction industry: An empirical study in China. J. Clean. Prod..

[23] Niu S., Sun G., Yang G. (2024). Distributionally robust optimization for a capacity-sharing supply chain network design problem. J. Clean. Prod..

[24] Duan K., Pang G., Lin Y. (2023). Exploring the current status and future opportunities of blockchain technology adoption and application in supply chain management. J. Digit. Econ..

[25] Kara M.E., Fırat S.Ü.O., Ghadge A. (2020). A data mining-based framework for supply chain risk management. Comput. Ind. Eng..

[26] Chung S.H., Sah B., Lee J. (2020). Optimization for drone and drone-truck combined operations: A review of the state of the art and future directions. Comput. Oper. Res..

[27] Li H., Chen J., Wang F., Bai M. (2021). Ground-vehicle and unmanned-aerial-vehicle routing problems from two-echelon scheme perspective: A review. Eur. J. Oper. Res..

[28] Luo Z., Poon M., Zhang Z., Liu Z., Lim A. (2021). The multi-visit traveling salesman problem with multi-drones. Transp. Res. Part C Emerg. Technol..

[29] Diaz R., Kolachana S., Falcão Gomes R. (2023). A simulation-based logistics assessment framework in global pharmaceutical supply chain networks. J. Oper. Res. Soc..

[30] Nabayiga H., Van Der Meer R., Agha M.S.A. (2025). A systematic review of simulation models in medicine supply chain management: Current state and emerging trends. Decis. Anal. J..

[31] Jang J., Lee H. (2024). Effective hydrogen supply chain management framework considering nonlinear multi-stage process uncertainties. Appl. Energy.

[32] Peng J., Chen L., Zhang B. (2022). Transportation planning for sustainable supply chain network using big data technology. Inf. Sci..

[33] Matenga A.E., Mpofu K. (2022). Blockchain-based cloud manufacturing SCM system for collaborative enterprise manufacturing: A case study of transport manufacturing. Appl. Sci..

[34] Zulqarnain R.M., Naveed H., Siddique I., Alcantud J.C.R. (2024). Transportation decisions in supply chain management using interval-valued q-rung orthopair fuzzy soft information. Eng. Appl. Artif. Intell..

[35] Moh’d Anwer A.-S. (2022). An investigation of transportation logistics strategy on manufacturing supply chain responsiveness in developing countries: The mediating role of delivery reliability and delivery speed. Heliyon.

[36] Chen Q., Miller-Hooks E., Huang E. (2023). Assessing transportation infrastructure impacts from supply chain restructuring for increased domestic production of critical resources. Comput. Ind. Eng..

[37] Zaroni, Musari K. (2020). Blockchain, Digitalisasi Logistik menuju Halal Global. TruckMagz.

[38] Bux C., Varese E., Amicarelli V., Lombardi M. (2022). Halal food sustainability between certification and blockchain: A review. Sustainability.

[39] Khaleghi M., Sheykhivand S., Khaleghi N., Danishvar S. (2026). An Intelligent Multi-Task Supply Chain Model Based on Bio-Inspired Networks. Biomimetics.

[40] Ab Talib M., Pang L., Md Said N. (2021). What Can the Brunei Government Do to Encourage Halal Logistics Adoption: Lessons from the Literature. Oper. Supply Chain Manag. An. Int. J..

[41] Bassiouni M.M., Chakrabortty R.K., Sallam K.M., Hussain O.K. (2024). Deep learning approaches to identify order status in a complex supply chain. Expert Syst. Appl..

[42] Ngah A.H., Zainuddin Y., Thurasamy R. (2015). Barriers and enablers in adopting of Halal warehousing. J. Islam. Mark..

[43] Wang K., Zhao Y., Gangadhari R.K., Li Z. (2021). Analyzing the adoption challenges of the Internet of things (Iot) and artificial intelligence (ai) for smart cities in china. Sustainability.

[44] Akbari-Aghghaleh Z., Mozdgir A., Seyedi I., Messina E. (2025). Designing a perishable closed-loop poultry supply chain: Metaheuristic approaches and model evaluation. Environ. Dev. Sustain..

[45] Tsolaki K., Vafeiadis T., Nizamis A., Ioannidis D., Tzovaras D. (2023). Utilizing machine learning on freight transportation and logistics applications: A review. ICT Express.

[46] Deng M., Bian B., Zhou Y., Ding J. (2023). Distributionally robust production and replenishment problem for hydrogen supply chains. Transp. Res. Part E Logist. Transp. Rev..

[47] Fartaj S.-R., Kabir G., Eghujovbo V., Ali S.M., Paul S.K. (2020). Modeling transportation disruptions in the supply chain of automotive parts manufacturing company. Int. J. Prod. Econ..

[48] Defferrard M., Bresson X., Vandergheynst P. (2016). Convolutional neural networks on graphs with fast localized spectral filtering. Adv. Neural Inf. Process. Syst..

[49] Henaff M., Bruna J., LeCun Y. (2015). Deep convolutional networks on graph-structured data. arXiv.

[50] Khaleghi N., Hashemi S., Ardabili S.Z., Sheykhivand S., Danishvar S. (2023). Salient Arithmetic Data Extraction from Brain Activity via an Improved Deep Network. Sensors.

[51] Monti F., Boscaini D., Masci J., Rodola E., Svoboda J., Bronstein M.M. Geometric deep learning on graphs and manifolds using mixture model cnns. Proceedings of the IEEE Conference on Computer Vision and Pattern Recognition.

[52] Velickovic P., Cucurull G., Casanova A., Romero A., Lio P., Bengio Y. (2017). Graph attention networks. Stat.

[53] Straka M., Kleinová K. (2026). Deep Learning-Enhanced Proactive Strategy: LSTM and VRP/ACO for Autonomous Replenishment and Demand Forecasting in Shared Logistics. Appl. Sci..

[54] Katangoori A. (2026). An Empirical Analysis of Data-Driven Supply Chain Optimization in Retail and Logistics. Supply Chain. Anal..

[55] Wasi A.T., Islam M., Akib A.R., Bappy M.M. (2024). Graph Neural Networks in Supply Chain Analytics and Optimization: Concepts, Perspectives, Dataset and Benchmarks. arXiv.

[56] Mucherino A., Papajorgji P.J., Pardalos P.M., Mucherino A., Papajorgji P.J., Pardalos P.M. (2009). K-nearest neighbor classification. Data Min. Agric..

[57] Chen T., Guestrin C. Xgboost: A scalable tree boosting system. Proceedings of the 22nd ACM Sigkdd International Conference on Knowledge Discovery and Data Mining.

[58] Xu K., Hu W., Leskovec J., Jegelka S. (2018). How powerful are graph neural networks?. arXiv.

[59] Yan T., Wan Z., Zhang P. Fully transformer network for change detection of remote sensing images. Proceedings of the Asian Conference on Computer Vision.

[60] Tavana M., Saberi E., Dooz A.P., Mina H. (2026). A Multi-Depot Vehicle Routing Optimization Model for Quick Commerce Last-Mile Delivery. Electron. Commer. Res. Appl..

[61] Gheitarani F., Ravanbeh S., Abdoli N., Yousefi F., Goldarzehi R., Atrian A. (2024). Categorization of Blockchain Technology Applications in Human Resource Management: An Interpretive Structural Modeling Approach. SSRN Electron. J..

[62] Jadidi V., Ardakani H.T., Hanif H.R., Naseri S.Z. (2024). Examining How New Technologies Affect Management and Decision-Making Processes in Organizations. Adv. J. Manag. Humanit. Soc. Sci..

[63] Rahmani M., Mohajelin F., Khaleghi N., Sheykhivand S., Danishvar S. (2024). An Automatic Lie Detection Model Using EEG Signals Based on the Combination of Type 2 Fuzzy Sets and Deep Graph Convolutional Networks. Sensors.

[64] Sheykhivand S., Rezaii T.Y., Saatlo A.N., Romooz N. (2017). Comparison between Different Methods of Feature Extraction in BCI Systems Based on SSVEP. Int. J. Ind. Math..

[65] Bevilacqua C., Sohrabi P., Hamdy N. (2025). Integrating Ecosystem Services into Urban Carbon Dynamics: A Dual-Scale Spatial Analysis of Land Use, Emissions, and Planning. Land.

[66] Sohrabi P. (2023). Managing Urban Transition: Place-Sensitive Approach Towards Technological Resilience. Ph.D. Thesis.

[67] Dehnavi H.D., Azizi Y., Shafiei M. (2015). A New Method Based on Fuzzy System and Gravitational Optimal Detector for Capacitor Placement, Considering Nonlinear Loads. Proceedings of the 2015 30th International Power System Conference (PSC), Tehran, Iran, 23–25 November 2015.

[68] Jadidi V. (2025). The Impact of Artificial Intelligence on Judicial Decision-Making Processes. Adv. J. Manag. Humanit. Soc. Sci..

[69] Moghaddam P.K., Izadian N., Haghighatjoo M., Jafari A.M., Zahedi M. (2025). The Impact of Design Team Characteristics on Construction Project Performance with the Mediating Role of Construction Project Costs. Tech. J. (Teh. Glas.).

[70] Zarei M., Zarei O., Karimi M., Skandari M.R., Haghighatjoo M., Worya Khordehbinan M. (2024). The Application of Multi-Criteria Decision Analysis in Gaining a Premier Sort of Stability in Airplane Safety. Saf. Reliab..

[71] Ramey K.E., Velasquez A., Cheyney K., Beck M., Cota M., Schamberger B., Baradaran Shoraka Z. Culturally Revitalizing STEAM Learning as a Space for Ecologically Situated Identity Work. Proceedings of the 19th International Conference of the Learning Sciences (ICLS 2025).

[72] Perez G., Shrestha P., Cameron T., Waight N., Kayumova S., Rish R., Tripp J., Mozaffari F., Scheuneman S.M. The Role of Peer Interaction and Language Resources in Informal Engineering Learning Environments: The Case for Learning Through Biking. Proceedings of the 2025 ASEE Annual Conference & Exposition.

[73] Rish R.M., Waight N., Tripp J., Scheuneman S.M., Mozaffari F., Goehrig F., Jackson D.W., Kahveci E.N., Johnson G., Marks D.R. (2025). Mobilizing Youth STEM Learning Trajectories on Bicycles. Proceedings of the 19th International Conference of the Learning Sciences (ICLS 2025), Helsinki, Finland, 10–13 June 2025.

[74] Barati-Nia A. (2026). Characterizing the Effect of Plasticity Index on Monotonic and Cyclic Shear Behavior of Natural Low-Plastic Silt Mixtures. Ph.D. Dissertation.

[75] Parkami F., Lotfi A., Ragan K., Nelson M., Schipper J. (2026). Footsteps Through the Wild: Unraveling the Factors Behind Human Recreational Activities along the Arizona Trail, USA. J. Park Recreat. Adm..

[76] Villa S. (2022). Competing for supply and demand: Understanding retailers’ ordering decisions. Int. J. Prod. Econ..

[77] Torabi Z.-A., Hall C.M., Ravanbeh S., Zare N., Beiraghi Khatibi N. (2025). Unraveling Tourist Behavior in Tehran’s Rural Fringe: Moral Norms and Environmental Concerns in Shemiranat’s Biodiversity. J. Qual. Assur. Hosp. Tour..

[78] Mozaffari F., Ghodratinia Z. (2015). Extroversion and Introversion: The Effect of Teacher’s Personality on Elementary EFL Learners’ Achievement. IOSR J. Humanit. Soc. Sci..

[79] Khajehzadeh M., Pazhuheian F., Seifi F., Ghorbani A., Madraki G. (2024). A Novel Prescriptive Supply Chain Analytics Model for Monitoring the Relationship between Influential Variables across the Supply Chain Network. Oper. Supply Chain Manag..

[80] Jadidi V. (2025). Understanding Deviance: Social Norms and the Consequences of Nonconformity. Adv. J. Manag. Humanit. Soc. Sci..

[81] Jadidi V., Yazdani S., Mansour Z. (2025). Urban Violence and Social Psychology: From Causes to Prevention.

[82] Mohseni M., Faghihi R., Haghighatafshar M., Entezarmahdi S.M. (2018). Effects of the Attenuation Correction and Reconstruction Method Parameters on Conventional Cardiac Dynamic SPECT. Medicine.

[83] Hosseinidoust E., Sepehrdoost H., Khodabakhshi A., Massahi S. (2021). Investigating Interactions among Health Care Indicators, Income Inequality and Economic Growth: A Case Study of Iran. J. Appl. Econ. Stud. Iran..

[84] Geldi Geldi Nejad M. (2025). Pata and Diploma: Strategies for Sustaining Indigenous Knowledge Transmission in the Modern Music Schools of Turkmenistan. Asian Music.

[85] Hosseinzadeh G., Yousefi A., Sajjadi S.M., Vafaie R.H., Sheykhivand S., Ghazani M.S., Dabbagh J. (2025). Construction of TiO_2_ nanorod/graphene/Cu_3_P nanocomposite as indirect Z-scheme heterojunction photocatalyst for the treatment of oil refinery wastewater. Surf. Interfaces.

[86] Ahmadi B., Razi S., Saghand M.P., Changizi N., Fallah A.S., Tootkaboni M. (2024). Cold-Formed Cross-Sectional Folds with Optimal Signature Curve. J. Eng. Mech..

[87] Chang Y., Winkler A.J., Noori A., Knyazikhin Y., Myneni R.B. (2025). Precipitation Leads the Long-Term Vegetation Increase in the Conterminous United States Drylands. Environ. Res. Lett..

[88] Jamshidi S., Dehnavi A., Vaez Roudbari M., Yazdani M. (2024). An Integrated Approach through Controlled Experiment and LCIA to Evaluate Water Quality and Ecological Impacts of Irrigated Paddy Rice. Environ. Sci. Pollut. Res..

[89] Khashei Z. (2010). The Role of Passive Systems in Providing Comfort in Traditional Houses in Isfahan: A Case Study of the Karimi House. WIT Trans. Ecol. Environ..

[90] Karimi S.M., Hassani A.H., Zarei H., Moghadami M., Ali Parh M.Y., Shakib S.H., Aranha V., Mansouri M., Allen T., Chen Y. (2025). Geographical Pat-terns of COVID-19 Vaccine Inequality by Race and Ethnicity and Sociodemographic Determinants of Health: Evidence from Louisville, Kentucky. Vaccines.

[91] McCoy N.D., Gawrys S.P., Mackintosh S.G., Byrum S., Kosari N., Zhou A., Mansoor M.A.M., Ikeno Y., Isola J.V.V., Stout M.B. (2025). Ovarian Somatic Tissue Rejuvenates Circulating Apolipoproteins and Promotes Cognitive Health in Postreproductive Female Mice. GeroScience.

[92] Kosari N., Gustafson B., Zhou A. (2025). Probing Cytotoxic and Oxidative Stress Effects of Nanoplastics on Human Intestinal Caco-2 Cells: Insights from Raman Spectroscopy and Machine Learning. Microsc. Microanal..

[93] Kosari N., Mansourpour Z., Yazdian F. (2025). Extracting Kinetic Model for Natural Pigment Production from Monascus purpureus Using CFD Simulation. Eur. J. Eng. Technol. Res..

[94] Fennel Z.J., Kosari N., Bourrant P.-E., Yee E.M., Castro R.J., Kurian A.S., Palmer J., Christensen M., Funai K., O’Connell R.M. (2025). Macrophage Metabolic Rewiring Rejuvenates Muscle Raman Signatures and Cellular Remodeling during Regrowth in Aged Mice. JCI Insight.

[95] Motavaselian M., Bayati F., Amani-Beni R., Khalaji A., Haghverdi S., Abdollahi Z., Sarrafzadeh A., Manzelat A.M.R., Rigi A., Bahri R.A. (2022). Diagnostic Performance of Magnetic Resonance Imaging for Detection of Acute Appendicitis in Pregnant Women: A Systematic Review and Meta-Analysis. Arch. Acad. Emerg. Med..

[96] Reda A., Hasanzadeh A., Ghozy S., Sanjari Moghaddam H., Adl Parvar T., Motavaselian M., Kadirvel R., Kallmes D.F., Rabinstein A. (2025). Risk of Symptomatic Intracranial Hemorrhage after Mechanical Thrombectomy in Randomized Clinical Trials: A Systematic Review and Meta-Analysis. Brain Sci..

[97] Shamabadi A., Karimi H., Arabzadeh Bahri R., Motavaselian M., Akhondzadeh S. (2024). Emerging Drugs for the Treatment of Irritability Associated with Autism Spectrum Disorder. Expert Opin. Emerg. Drugs.

[98] Motavaselian M., Farrokhi M., Jafari Khouzani P., Moghadam Fard A., Daeizadeh F., Pourrahimi M., Mehrabani R., Amani-Beni R., Farrokhi M., Sarnaghy F.J. (2024). Diagnostic Performance of Ultrasonography for Identification of Small Bowel Obstruction: A Systematic Review and Meta-Analysis. Arch. Acad. Emerg. Med..

[99] Younesi Ramdani A., Alizadeh M.H., Minoonejad H., Emami Hashemii S.A. (2015). Comparison of the Static and Dynamic Balance of Female and Male Methadone-Maintained Opioid Dependents with Healthy Subjects. Sci. J. Rehabil. Med..

[100] Younesi Ramdani A., Alizadeh M.H., Minoonejad H., Emami Hashemi S.A. (2018). Comparison of the Spinal Posture in Sagittal Plane of Female and Male Methadone-Maintained Opioid Dependents with Healthy Subjects. J. Res. Sport Rehabil..

[101] Raeisi Z., Roshanzamir A., Abedi Lomer F., Ahmadi Lashaki R. (2026). YOLOv8 with Innovative Dilated Residual and Attention Modules for Mammographic Tumor Detection. Comput. Electr. Eng..

[102] Zare S., Raeisi Z., Ahmadi Lashaki R., Makki M. (2026). Design of a Thermoacoustic Engine Using Vanishing Perturbation and Genetic Algorithm. Results Eng..

[103] Khandan Khadem-Reza Z., Ahmadi Lashaki R., Shahram M.A., Zare H. (2025). Automatic Diagnosis of Autism Spectrum Disorders in Children through Resting-State Functional Magnetic Resonance Imaging with Machine Vision. Quant. Imaging Med. Surg..

[104] Abroumand Gholami A., Rahmani S., Moharreri P., Amirazodi E., Molavi A.M., Mokhtari T., Tahmasebi F., Rabiei Rad A., Babaloo H. (2025). Liposomal Ellagic Acid Enhances the Regenerative Potential of ADMSC-Laden Nanofibrous PCL Scaffolds in a Rat Model of Spinal Cord Injury. Sci. Rep..

[105] Nejadshamsi S., Bentahar J., Eicker U., Wang C., Jamshidi F. (2025). A geographic-semantic context-aware urban commuting flow prediction model using graph neural network. Expert Syst. Appl..

[106] Gholami A.A., Khachatryan L.G., Gulnoza R., Mortazavi F., Nilufar N., Tahmasebi F., Babaloo H., Davlatov S. (2026). Spinal Cord Injury as a Window into Hippocampal Dysfunction: Linking Inflammation, Neurogenesis, and Network Oscillations to Cognitive Decline. Exp. Neurol..

[107] Bahardoust M., Mousavi S., Dehkharghani M.Z., Arab M., Rashidi H., Gorgani H., Haghmoradi M., Askari A. (2024). Association of Tramadol Versus Codeine Prescriptions with All-Cause Mortality and Cardiovascular Diseases among Patients with Osteoarthritis: A Systematic Review and Meta-Analysis of Propensity Score-Matched Population-Based Cohort Studies. Adv. Rheumatol..

[108] Maadani M., Sarraf N.S., Alilou S., Aeinfar K., Sadeghipour P., Zahedmehr A., Fathollahi M.S., Ghadi S.I.H., Zavarehee A., Zolfaghari M. (2022). Relationship between Preprocedural Lipid Levels and Periprocedural Myocardial Injury in Patients Undergoing Elective Percutaneous Coronary Intervention. Tex. Heart Inst. J..

[109] Rahmani S.M., Faridaalaee G., Dehkharghani M.Z., Pouryahya P. (2020). Hyperkalaemia-Induced Narrow QRS Complex Complete Heart Block. Emerg. Med. Trauma Care J..

[110] Rahmani A., Soleymani A., Almukhtar M., Behzad Moghadam K., Vaziri Z., Hosein Tabar Kashi A., Adabi Firoozjah R., Jafari Tadi M., Zolfaghari Dehkharghani M., Valadi H. (2024). Exosomes and the Potential for Exosome-Based Interventions against COVID-19. Rev. Med. Virol..

[111] Awasthi S., Dehkharghani M.Z., Fudolig M. (2025). Emergence to Dominance: Estimating Time to Dominance of SARS-CoV-2 Variants Using Nonlinear Statistical Models. PLoS ONE.

[112] Bahardoust M., Dehkharghani M.Z., Ebrahimi P., Najafirashed M., Mousavi S., Haghmoradi M., Khaleghian M., Tizmaghz A. (2023). Effect of ABO Blood Group on Postoperative Overall Survival and Recurrence-Free Survival Rate in Patients with Hepatocellular Carcinoma after Hepatectomy: A Multi-Center Retrospective Cohort Study. BMC Surg..

[113] Pirzaman A.T., Sepidarkish M., Alizadeh F., Al-Obidy S., Ebrahimi P., Kianifard N., Nooshabadi M.S., Tadi M.J., Dehkharghani M.Z., Mousavi S. (2024). Prevalence of Human Schistosoma mansoni Infection in Endemic Regions (2010–2024): A Systematic Review and Meta-Analysis. eClinicalMedicine.

[114] Dehkharghani M.Z., Mousavi S., Kianifard N., Fazlzadeh A., Parsa H., Pirzaman A.T., Fazlollahpour-Naghibi A. (2024). Importance of Long Non-Coding RNAs in the Pathogenesis, Diagnosis, and Treatment of Myocardial Infarction. IJC Heart Vasc..

[115] Moarrefzadeh A., Sarandili S., Motamed-Gorji N., Majdolashrafi F., Bahardoust M., Mousavi S., Hashemi N., Sarveazad A., Vazirizadeh-Mahabadi M., Habibi S.A. (2025). Predictors of Quality of Life in Patients with Parkinson’s Disease: A Multicenter Case-Control Study. Basic Clin. Neurosci..

[116] Khatir A.A., Abbasi A., Sarandili S., Sepidarkish M., Fazlollahpour-Naghibi A., Arjmandi D., Rostami A. (2025). The Association between Parkinson Disease and Toxocara Infection/Exposure: A Case-Control Study. J. Helminthol..

[117] Sun Y. (2025). Financial Transaction Network Risk Prediction Model Based On Graph Neural Network. Procedia Comput. Sci..

[118] Dolatabadi A., Heydari M., Hashempour B., Asadiof F., Radmehr S. (2025). Linear and Nonlinear Analysis of Multimodal Physiological Data for Emotion Recognition. J. Artif. Intell. Syst. Model..

[119] Rezaei N., Mirtalebi Z. (2026). Quantifying the Impact of Data Cleaning on Deep Learning-Based Bearing Fault Diagnosis: A Multidataset, Multiarchitecture Study. Concurr. Comput. Pract. Exp..

[120] Yuan J., Pho K.H. (2023). Temperature Estimation of Smart Homes with Sensors in Internet of Things Environment Based on Blockchain. Adv. Eng. Intell. Syst..

[121] Subbarao B.V.S.S. (2023). Knowledge Management in Road Accident Detection Based on Developed Deep Learning. Adv. Eng. Intell. Syst..

[122] Yavuz S., Korkmaz E. (2023). Blockchain Approaches Survey for Big Data: Systematic Study, Challenges and Innovations. J. Artif. Intell. Syst. Model..

[123] Lu X. (2025). Detecting Depression via Tweets on Twitter Utilizing Machine Learning and Neural Network Models. J. Artif. Intell. Syst. Model..

[124] Saremi S. (2025). The Strategic Role of Information Systems in Modern Business: Empowering Decision-Making and Sustaining Competitive Advantage. Proceedings of the International Conference on Science, Engineering Management and Information Technology, Dubai, United Arab Emirates, 11–13 September 2025.

[125] Koushyar J.M., Guirguis M., Atia G. (2025). PuRe Defender: A Game-Theoretic Pull Request Assignment with Deep RL. Proceedings of the International Conference on Game Theory and AI for Security, Athens, Greece, 13–15 October 2025.

[126] Rivandi E., Oskouei R.J. (2025). A Novel Approach for Developing Intrusion Detection Systems in Mobile Social Networks. J. Soft Comput. Decis. Anal..

[127] Bagherabad M.B., Rivandi E., Mehr M.J. (2026). Machine Learning for Analyzing Effects of Various Factors on Business Economic. Appl. Decis. Anal..

[128] Rivandi E. (2026). FinTech and the Level of Its Adoption in Different Countries around the World. Manag. Sci. Adv..

[129] Pezeshgi A., Naeimi M., Family Q. (2025). Buying on Impulse in the Age of AI: Mechanisms, Evidence, and Moral Dilemmas. SSRN Electron. J..

[130] Pezeshgi A., Abarghoei M.V., Naeimi M., Family Q. (2026). Trusting the Machine: How Consumer Trust in Artificial Intelligence Shapes Future Adoption Intentions. Manag. Sci. Adv..

[131] Fathi N. (2026). EDFNet: Early Fusion of Edge and Depth for Thin-Obstacle Segmentation in UAV Navigation. arXiv.

[132] Mahpouya F., Burris C.J., Paul H., Nikolaev A. (2026). Maximizing the Expected Value of Experimentation for Finding Top-κ Rank via Aggregation of Pairwise Comparisons. IISE Trans..

[133] Li B., Karami M., Junayed M.S., Nabavi S. (2024). Multi-Modal Spatial Clustering for Spatial Transcriptomics Utilizing High-Resolution Histology Images. Proceedings of the 2024 IEEE International Conference on Bioinformatics and Biomedicine (BIBM), Lisbon, Portugal, 3–6 December 2024.

[134] Mansouri S., Samatova V., Korchiev N., Anyanwu K. (2023). DeMaTO: An Ontology for Modeling Transactional Behavior in Decentralized Marketplaces. Proceedings of the 2023 IEEE/WIC International Conference on Web Intelligence and Intelligent Agent Technology (WI-IAT), Venice, Italy, 26–29 October 2023.

[135] Mansouri S., Mohammed H., Korchiev N., Anyanwu K. (2024). Taming Smart Contracts with Blockchain Transaction Primitives: A Possibility?. Proceedings of the 2024 IEEE International Conference on Blockchain, Copenhagen, Denmark, 19–22 August 2024.

[136] Korchiev N., Pateria A., Samatova V., Mansouri S., Anyanwu K. (2024). Taming the Beast of User-Programmed Transactions on Blockchains: A Declarative Transaction Approach. arXiv.

[137] Anyanwu K., Mansouri S., Adei D. (2025). Towards Declarative Blockchains: A SHACL-Based Model for Robust and Efficient Transactions. Proceedings of the 2025 IEEE International Conference on Blockchain and Cryptocurrency (ICBC), Pisa, Italy, 2–6 June 2025.

[138] Samatova V., Korchiev N., Mansouri S., Anyanwu K. (2024). Towards a Smart Asset Model for Digital Assets on Blockchains. Proceedings of the 2024 IEEE/WIC International Conference on Web Intelligence and Intelligent Agent Technology (WI-IAT), Bangkok, Thailand, 9–12 December 2024.

[139] Amiri M.K., Zaferani S.P.G., Emami M.R.S., Zahmatkesh S., Pourhanasa R., Namaghi S.S., Klemeš J.J., Bokhari A., Hajiaghaei-Keshteli M. (2023). Multi-Objective Optimization of Thermophysical Properties GO Powders-DW/EG NF by RSM, NSGA-II, ANN, MLP and ML. Energy.

[140] Vaghfi Mohebbi P., Lu Y., Miao Z., Balasundaram B., Kalgotra P., Sharda R. (2025). Identifying Most Lethal Cliques in Disease Comorbidity Graphs. IISE Trans. Healthc. Syst. Eng..

[141] ArfaeiYazdiPour M., Zargari F., Samimi A., Sahebi S. (2026). Analyzing the Influence of Fuel Price Shock on Urban Public Transit and Interurban Automobile Travel Demand: Evidence from a Country with Fixed Fuel Price Regulation. Transp. Res. Interdiscip. Perspect..

[142] Asgharpour S., Allahyari A., ArfaeiYazdiPour M., Mohammadian A.K. (2026). How Racial Segregation Contributes to Disparities in Pedestrian Safety. J. Saf. Res..

[143] Safizadeh M., Yazdanparast A., Felix R. (2026). Taking Pride in Vegan Consumption: A Construal Level Theory Account of Ad Message Appeal and Future Self Connectedness. Psychol. Mark..

[144] Roshdieh N. (2024). The Effect of Monetary Policy Uncertainty on Stock Market Uncertainty with NARDL Approach. Res. J. Financ. Account..

[145] Obenauer W., Rezaei S. (2023). #MeToo, COVID-19 and the New Workplace: Re-Examining Institutional Discrimination’s Impact on Workplace Harassment of Expatriates Following Two Exogenous Shocks. J. Glob. Mobil..

[146] Pourghasemi M., Rezaei E., Ansariyar A., Ardeshiri A., Jeihani M., Tootkaboni M. (2026). Driving Behavior Recognition and Scoring: A Bayesian Approach to Driving Simulator Data Analytics. Accid. Anal. Prev..

[147] Bevilacqua C., Hamdy N., Sohrabi P. (2025). Linking Land Uses and Ecosystem Services through a Bipartite Spatial Network: A Framework for Urban CO_2_ Mitigation. Sustainability.

[148] Bevilacqua C., Vitiello G., Sebillo M.M.L., Provenzano V., Sohrabi P., Hamdy N., Trapani F., Pizzimenti P. A Multidisciplinary Approach to Plan ECOsystem SErvices for Cities in Transition. Proceedings of the 16th Biannual Conference of the Italian SIGCHI Chapter.

